# Isostructurality and energetics of competitive C–H⋯π interactions in adamantane-benzoate hybrids: a computational ADME study

**DOI:** 10.1039/d6ra01672c

**Published:** 2026-06-02

**Authors:** Hiram Pérez

**Affiliations:** a Universidad de La Habana, Facultad de Química Zapata s/n, e/ G y Carlitos Aguirre Vedado Habana 10400 Cuba hiramperezperez49@gmail.com

## Abstract

A comprehensive investigation of the supramolecular architecture in nine 2-(adamantan-1-yl)-2-oxoethyl benzoates is reported, integrating experimental X-ray data with advanced theoretical analysis. Beyond expanding on previously reported structural aspects, this work quantifies isostructural relationships using the dissimilarity index (*X*) and packing similarity (*PS*_ab_), providing a rigorous numerical basis for observed packing motifs. Crystal stability is governed by a complex interplay of C–H⋯O hydrogen bonds and “edge-to-face” C–H⋯π contacts, further supported by C–H⋯Cl interactions and π-stacking in specific derivatives. Lattice and interaction energies were partitioned using the PIXEL method. Visualization and quantification of the intermolecular contacts were performed by Hirshfeld surface analysis. The electronic nature and cooperativity of the adamantane–benzoate C–H⋯π contacts were elucidated through MEP, QTAIM-NCI and NBO calculations. Finally, the pharmacological potential of the series was evaluated *via in silico* ADME predictions. The compounds exhibited high gastrointestinal absorption and optimal drug-likeness parameters, although their high lipophilicity suggests potential limitations in aqueous solubility. These results characterize the adamantane-benzoate scaffold as a viable candidate for future drug design.

## Abstract 2

This study quantifies isostructurality, Pixel energy, Hirshfeld surfaces, unraveling the role of methine/methylene protons in H⋯π contacts of adamantane-benzoate hybrids *via* DFT. ADME analysis predicts good pharmacokinetic potential.

## Graphical abstract

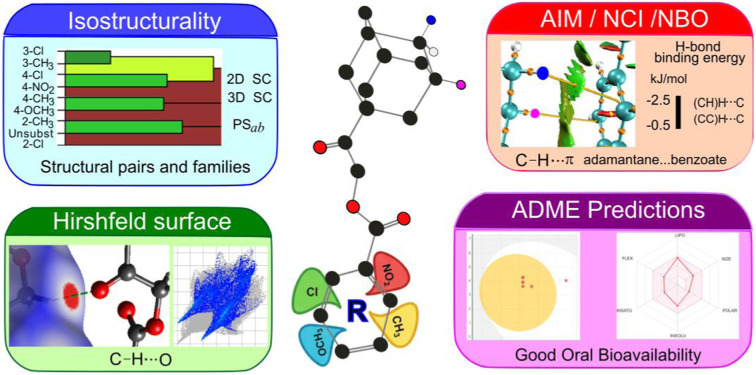

## 1 Introduction

Recent advances in drug discovery have underscored the pharmacological relevance of adamantane- and benzoate-based compounds. The rigid, hydrophobic cage of adamantane enhances metabolic stability, lipophilicity, and receptor binding affinity.^1^ The synthesis of these hybrids has evolved from early phase-transfer catalysis methods, which demonstrated enhanced analgesic activity,^2,3^ to contemporary metal-free cascade protocols for modular assembly.^4,5^ Despite their importance, comprehensive investigations focusing on the energetics of supramolecular assembly and the reevaluation of isostructurality in adamantane-benzoate hybrids remain scarce.

An interesting systematic study was reported in order to understand the mode of interaction of adamantane with benzene, unraveling the propensity of methine and methylene C–H groups of adamantane to participate in C–H⋯π interactions, by using both *ab initio* and DFT methods.^6^

In a more recent study,^7^ sixteen adamantane-based oxoethyl benzoates (hereafter AOEB) and thirty-six phenacyl benzoate derivatives were compared to evaluate the effect of replacing the electron-rich phenyl ring with the bulky adamantane moiety on molecular conformation and crystal packing. However, that study did not provide a detailed characterization of the non-covalent interactions within the AOEB system, merely suggesting that the adamantane cage reduces the occurrence of weak intermolecular π⋯π and C–H⋯π interactions relative to phenacyl benzoates. Furthermore, although high isostructurality was observed and attributed to constraints in packing patterns, a quantitative analysis of this phenomenon was not pursued.

**Scheme 1:**
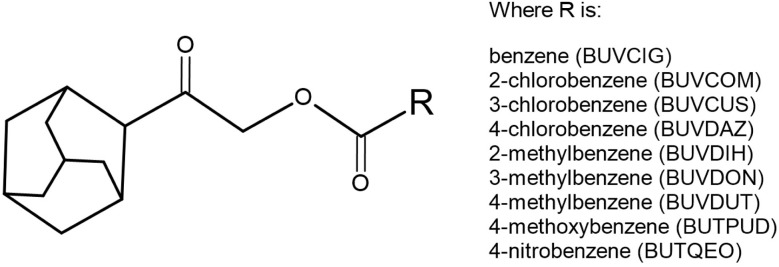
General structure of the studied adamantane-based oxoethyl benzoates (AOEB) and the list of R-substituted derivatives with their corresponding CSD codes.

Driven by the potential of this drug scaffold system, combining adamantane and benzoate moieties, a comprehensive analysis of nine AOEB is presented here (Scheme 1). These compounds correspond to those for which isostructural relationships were previously suggested,^7^ and feature various electron-donating and electron-withdrawing substituents on the benzene ring. Although the isostructurality remains a subject of active debate,^8^ this series provides an ideal platform for understanding of structural analogies, which is crucial in crystal engineering.^9^

Building upon these considerations, isostructurality is evaluated using geometric descriptors. This is complemented by Hirshfeld surface analysis and PIXEL energies. Additionally, theoretical methods were used to explore the electronic nature of C–H⋯π interactions between adamantane and benzoate groups, marking the first investigation of this motif within experimental structures.

Finally, as part of an ongoing interest in structure–activity relationship studies,^10^ a detailed insight into the potential of this series is provided through *in silico* ADME predictions. In this context, it is worth noting that these hybrids have shown inhibitory activity against acetylcholinesterase (AChE) and butyrylcholinesterase (BChE), primarily through hydrogen bonding and halogen interactions.^11^ Building upon this, the present work aims to provide a deeper understanding of the supramolecular assembly by focusing on the energetics of competitive C–H⋯π interactions, which play a crucial role in their structural stabilization and molecular recognition.

## 2 Methods and computational details

A comprehensive suite of computational tools was employed to explore the supramolecular assembly, energetic stability, and pharmacokinetic potential of the compounds, as described in the following subsections.

### 2.1 Crystal structure measurement

Geometrical calculations and structural analyses were performed using PLATON^12^ and Mercury (version 2024.3.1).^13^ Molecular illustrations and ORTEP diagrams were generated using ORTEP-3 for Windows (version 2023.1) within the WinGX software suite.^14^

### 2.2 Description of isostructurality

Quantitative assessment of isostructurality was performed using two complementary geometric approaches:

(a) Dissimilarity index ‘*X*’: calculations were carried out with XPac 2.0.2,^15^ a software designed to compare representative lists of internal coordinates distances, intermolecular angles, dihedral angles and torsion angles. The Xpac method allows the identification of similar packing arrangements present between two crystal structures.^16^ Common structural motifs present in crystal structures to be compared are termed as “supramolecular constructs” (SCs), which represent sub-components of complete crystal structures. The SC may be 0D similarity, 1D similarity (row of molecules match), 2D similarity (layer of molecules match) and 3D similarity (isostructural). Xpac defines the dissimilarity index *‘X’* as a measure of how far the two crystal structures deviate from perfect geometrical similarity.^17,18^ The lowest value for *X* indicates the highest degree of similarity. *X* values smaller than 1 are found for SCs with high similarity, whereas SCs of low-degree similarity produce *X* values of 6 or even higher.

(b) Packing similarity PS_ab_: computed *via* CrystalCMP,^19^ this descriptor facilitates comparing molecular packing and identifying identical motifs. Similarity is derived from relative positions and rotations of molecules within a representative molecular cluster; its low sensitivity to volume changes is ideal for comparing related compounds. The resulting transformation matrices were used to generate dendrograms for the hierarchical classification of molecular packing patterns.

### 2.3 Lattice and interaction energies

Lattice and intermolecular interaction energies were calculated using the PIXEL method as implemented in the CLP software package.^20^ This method allows for the partitioning of the total interaction energy (*E*_tot_) into coulombic (*E*_coul_), polarization (*E*_pol_), dispersion (*E*_disp_), and repulsion (*E*_rep_) components. For all calculations, the molecular geometries obtained from the X-ray crystal structures were used, with C–H distances normalized to standard neutron diffraction values to ensure reliable electrostatic and dispersion contributions. Accurate electron densities around the molecules were calculated at MP2/6-31G** level using Gaussian16.^21^ Additionally, the interaction energy tool Charger of MoProViewer^22^ was utilized to compute the total electrostatic energy *E*^elec^_tot_ of specific intermolecular contacts. *E*^elec^_tot_ includes two terms, the electrostatic interaction permanent *E*^elec^_perm_ and the polarization contribution *E*^elec^_pol_. Charger contains an implementation of the analytical computation of the electrostatic interaction energy (aEP/MM) method,^23^ allowing for highly accurate calculations at short interaction distances between any two selections of atoms.

### 2.4 Hirshfeld surfaces and enrichment ratios

Hirshfeld surfaces were generated using CrystalExplorer 21.5 (ref. 24) to provide detailed insights into the weak intermolecular interactions governing the crystal packing.^25,26^ The surfaces were mapped using *d*_norm_ and shape-index properties. The latter is a qualitative measure highly sensitive to subtle topological changes, particularly in planar regions. Complementary “bumps” and “hollows” on the surface are represented by shape indices of differing signs; specifically, an intermolecular interaction donor is associated with a blue “bump” (shape-index > 0), while a red “hollow” (shape-index < 0) represents the acceptor.

To identify favored intermolecular contacts, enrichment ratios (*E*_XY_)^27,28^ were computed using MoProViewer.^22^ This ratio for a pair of chemical elements (*X*, *Y*) is defined as the ratio between the proportion of actual crystal contacts and the theoretical proportion of equiprobable random contacts. The proportion *S*_X_ of each chemical species on the molecular surface is derived from the percentage contributions of the actual contact types, as calculated *via* the Hirshfeld surface approach. *E*_XY_ values greater than unity signify a high propensity for the elements to form contacts within the crystal structure, whereas values lower than unity indicate a tendency to avoid such interactions.

### 2.5 Theoretical DFT methods

Theoretical study of non-covalent interactions was performed at the B3LYP/6-31G(d,p) level of theory using Gaussian 16.^21^ Grimme's D3 dispersion correction was applied to accurately capture dispersion effects. All calculations were processed with Multiwfn^29^ and visualized using VMD^30^ and Avogadro.^31^

To evaluate the electrostatic complementarity between the monomers, Molecular Electrostatic Potential (MEP) maps were first generated on the *ρ*(*r*) = 0.001 a.u. electron density isosurface. The potential minima *V*_s,min_ (red regions) and maxima *V*_s,max_ (blue regions) were located and quantified following the framework established by Politzer *et al.*^32^ for characterizing non-covalent recognition sites.

Subsequently, two distinct structural models were prepared for the interaction analysis: (a) a crystallographic model generated by extracting the relevant molecular dimer directly from the experimental CIF data, and (b) two optimized models (for methylene and methine hydrogen interactions). Initial dimer geometries were manually adjusted to describe H⋯Cg (centroid) interactions with a starting distance of 3.0 Å and a *γ* angle of 0°. These specific configurations were subsequently optimized to ensure the identification of local minima on the potential energy surface. The dimerization energy (Δ*E*) was computed as the energy difference between the complex and the isolated monomers following the supermolecule approximation, including the Boys and Bernardi counterpoise correction.^33^

Non-covalent interactions were further characterized by a combined use of quantum theory of atoms in molecules (QTAIM)^34^ and NCIplot isosurfaces.^35^ QTAIM analysis enabled the identification of bond critical points (BCPs) and bond paths, while NCIplot provided complementary visualization based on the reduced density gradient (RDG). The isosurface color scheme follows a red–yellow–green–blue gradient where red indicates strong repulsion, blue indicates strong attraction, and green represents weak interactions. H-bond binding energies (BE) were estimated using the correlation established for neutral complexes by Emamian *et al.*^36^ [eqn (1)]:

1 BE (kcal mol^−1^) = −223.08·*ρ*_BCP_ + 0.3427

Finally, Natural Bond Orbital (NBO) analysis^37^ was carried out to evaluate the hyperconjugative charge transfer between donor and acceptor orbitals, providing a quantitative measure of the second-order perturbation energies (*E*^(2)^) involved in specific non-covalent contacts.

### 2.6 *In silico* ADME predictions

SwissADME is a web-based tool developed by Swiss Institute of Bioinformatics to evaluate characteristics of absorption, distribution, metabolism, and excretion (ADME).^38,39^ This tool allows predicting physicochemical properties, lipophilicity, water solubility, pharmacokinetics, drug-likeness, and medicinal chemistry suitability of small molecules, which represent critical parameters for the early stages of rational drug discovery.

The drug-likeness was also evaluated by checking if each compound complies with Lipinski's rule-of-five framework,^40^ which is a key predictor of oral bioavailability and potential pharmacological activity. Lipinski's rule states that, in general, an orally active drug exhibits good bioavailability if it has no more than one violation of the following criteria: (a) no more than 5 hydrogen bond donors (total N–H and O–H bonds); (b) no more than 10 hydrogen bond acceptors (N or O atoms); (c) a molecular mass (*MW*) less than 500 Da; and (d) a calculated partition coefficient (log *P*_o/w_) that does not exceed a value of 5.0.

This rule is a rule of thumb, and hence it has many exceptions. For this reason, additional extensions such as Ghose,^41^ Veber,^42^ Egan,^43^ and Muegge^44^ scores are considered, alongside the Bioavailability Score, which must be 0.55 to perform as a viable oral drug.^45^ Gastrointestinal absorption (GI) and Blood–Brain Barrier (BBB) permeation are two pharmacokinetic behaviors essential to estimate at various stages of the drug discovery processes. The data outputted by SwissADME exhibits a permeation graph named BOILED-Egg (Brain Or IntestinaL EstimateD),^46^ which predicts if a small molecule will be passively absorbed in the gastrointestinal tract (white area) or cross the blood–brain barrier (yellow yolk area), by plotting lipophilicity (described by the atomistic method *W* LOG *P*)^47^*versus* apparent polarity TPSA.^48^

If a plotted molecule falls inside the white ellipse, the probability of a good intestinal absorption is high. If it falls inside the yellow ellipse (the yolk), the probability of a good BBB crossing is high. Additionally, points colored in blue correspond to molecules predicted to be effluated by the central nervous system by the P-glycoprotein (*P*-gp^+^), and those in red are predicted not to be effluated (*P*-gp^−^). The output data also exhibits a Bioavailability Radar, which represents the suitable physicochemical space for oral bioavailability with the following parameters:

• LIPO (Lipophilicity): −0.7 < *X* LOG *P*3 < + 5.0 (ref. 47)

• SIZE: 150 g mol^−1^ < MW < 500 g mol^−1^.

• POLAR (Polarity): 20 Å^2^ < TPSA < 130 Å^2^

• INSOLU (Insolubility): −6 < log *S* (ESOL) < 0 (ref. 47)

• INSATU (Insaturation): 0.25<Fraction *C*_sp^3^_ < 1 (ref. 39)

• FLEX (Flexibility): 0 < Num. rotatable bonds < 9.

The log *S* scale is defined as: Insoluble < − 10 < Poorly < − 6 < Moderately < − 4 < Soluble < − 2 < Very <0< Highly.

## 3 Results and discussion

The structural and physicochemical properties of the selected benzoates were evaluated through a multi-analytical approach. This section begins with a detailed description of the molecular and crystal structures, providing the basis for a quantitative assessment of their isostructural relationships. To further understand the supramolecular assembly, lattice energy calculations, and a comprehensive Hirshfeld surface analysis were performed, complemented by a specific theoretical study of the adamantane⋯benzoate interactions. Finally, the series was evaluated using SwissADME predictions to establish a preliminary profile of their pharmacokinetic potential.

### 3.1 Molecular structures

**Fig. 1:**
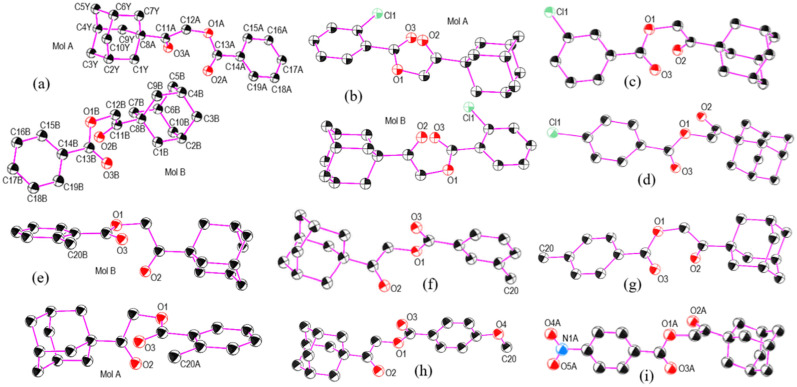
Molecular structures of nine 2-(adamantan-1-yl)-2-oxoethyl benzoates (AOEB): (a) BUVCIG, (b) BUVCOM, (c) BUVCUS, (d) BUVDAZ, (e) BUVDIH, (f) BUVDON, (g) BUVDUT, (h) BUTPUD, and (i) BUTQEO. Atom labeling is shown for (a); for the remaining structures, only relevant heteroatoms and substituted positions are highlighted for clarity.

A series of nine 2-(adamantan-1-yl)-2-oxoethyl benzoate derivatives (AOEB) was selected for this study due to their documented structural similarities. These compounds, originally reported in the literature,^7^ are identified by their respective Cambridge Structural Database (CSD) Refcodes: BUVCIG (unsubstituted), BUVCOM (2-Cl), BUVCUS (3-Cl), BUVDAZ (4-Cl), BUVDIH (2-Me), BUVDON (3-Me), BUVDUT (4-Me), BUTPUD (4-OMe), and BUTQEO (4-NO_2_). The molecular structures of these nine compounds are illustrated in Fig. 1. The crystallographic data were retrieved from the CSD, and all C–H bond distances were normalized to a standard neutron diffraction value (1.080 Å) to ensure consistency in the subsequent topological and energy analyses.

### 3.2 Packing similarity analysis

Crystallographic unit cell parameters and space group symmetries for the AOEB series (Table 1) indicate diverse degrees of packing similarity between specific pairs, such as BUVCIG/BUVDIH, BUVDAZ/BUTQEO, BUVDUT/BUTPUD, and BUVCUS/BUVDON. While some of these relations were previously noted,^7^ the original study suggested 2D similarity among BUVCUS, BUVDAZ, BUVDON, and BUTQEO without recognizing that the pairs BUVDAZ/BUTQEO and BUVCUS/BUVDON show 3D similarity.

**Table 1:** Unit cell parameters (Å, °) and space group (SG) for the nine AOEB compounds

Refcode	*a* (Å)	*b* (Å)	*c* (Å)	*α* (°)	*β* (°)	*γ* (°)	*V* (Å^3^)	*Z*′	SG
BUVCIG	9.709(1)	10.122(1)	17.759(2)	77.0	77.9	71.7	1596.0	4	*P*1̄
BUVCOM	9.917(1)	26.061(4)	13.675(2)	90.0	109.4	90.0	3332.8	4	*P*2_1_/*c*
BUVCUS	15.027(1)	6.432(1)	19.414(1)	90.0	118.8	90.0	1644.3	4	*P*2_1_/*c*
BUVDAZ	12.778(2)	6.489(1)	19.849(3)	90.0	90.3	90.0	1645.7	4	*P*2_1_/*c*
BUVDIH	9.721(1)	10.010(1)	18.954(1)	75.9	84.6	73.0	1710.4	2	*P*1̄
BUVDON	14.881(3)	6.439(1)	18.255(3)	90.0	108.0	90.0	1663.5	4	*P*2_1_/*n*
BUVDUT	8.906(1)	6.456(1)	29.368(3)	90.0	97.6	90.0	1673.7	4	*P*2_1_/*n*
BUTPUD	9.491(1)	6.480(1)	28.343(3)	90.0	99.1	90.0	1721.3	4	*P*2_1_/*n*
BUTQEO	12.882(1)	6.485(1)	20.003(1)	90.0	90.6	90.0	1670.8	4	*P*2_1_/*c*

To quantitatively evaluate these relationships considering the crystalline environment, we calculated the dissimilarity index *X* for ten molecular pairs.^49^ The Corresponding Ordered Sets of Points (COSP) were defined using a core of 22 non-H atoms from the adamantyl oxoethyl benzoate moiety, with the exception of the BUVCUS/BUTQEO pair, which required a set of 20 non-H atoms. The primary XPac results are summarized in Table 2. A view of the outputted results by XPac for all the compounds are shown in Fig. S1–S10. The 3D supramolecular constructs (3D SC) for BUVCUS/BUVDON, BUVDAZ/BUTQEO, BUVCIG/BUVDIH, and BUVDUT/BUTPUD indicate *X* values of 1.6, 2.8, 6.4, and 6.7, respectively, revealing moderate 3D similarity (1.0 < *X* < 6.0) for the first two, and lower 3D similarity (*X* > 6.0) for the latter. Furthermore, moderate 2D similarity was identified for the pairs BUVDON/BUTQEO (*X* = 3.5), BUVCUS/BUTQEO (*X* = 4.4), BUVDAZ/BUVDON (*X* = 4.9), and BUVCUS/BUVDAZ (*X* = 5.1). In contrast, the pairs BUVCOM/BUVCIG and BUVCOM/BUVDIH exhibit low 2D similarity, with *X* values of 6.9 and 8.4, respectively.

**Table 2:** XPac analysis results for selected AOEB molecular pairs

Molecular pair	*n* a	Δ[*a*]^b^	Δ[*p*]^c^	SC^d^	*X* e	*D* f
BUVCUS/BUVDON	14	0.7	1.4	3D	1.6	0.09
BUVDAZ/BUTQEO	14	1.1	2.5	3D	2.8	0.15
BUVDUT/BUTPUD	14	3.1	6.0	3D	6.7	0.37
BUVCIG/BUVDIH	21	3.0	2.5	3D	6.4	0.29
BUVDON/BUTQEO	6	1.6	3.1	2D	3.5	0.06
BUVCUS/BUTQEO	6	2.4	3.7	2D	4.4	0.15
BUVDAZ/BUVDON	6	2.1	4.4	2D	4.9	0.06
BUVCUS/BUVDAZ	6	2.4	4.5	2D	5.1	0.07
BUVCOM/BUVCIG	19	3.6	7.5	2D	8.4	0.31
BUVCOM/BUVDIH	23	2.5	6.3	2D	6.9	0.14

a *n*: number of neighbors.

b Δ[*a*]: mean deviation in angles (°).

c Δ[*p*]: mean deviation in planes (°).

d SC: supramolecular construct.

e *X*: dissimilarity index.

f *D*: stretch parameter (Å).

By the other side, the occurrence of 2D supramolecular constructs (2D SC) with *X* values of 3.5, 4.4, 4.9 and 5.1 for the pairs BUVDON/BUTQEO, BUVCUS/BUTQEO, BUVDAZ/BUVDON, and BUVCUS/BUVDAZ indicate moderate 2D structural similarity, whereas *X* values of 6.9 and 8.4 denote low 2D similarity for BUVCOM/BUVCIG and BUVCOM/BUVDIH (Table 2).

Finally, the stretch parameter *D* in the range 0.06–0.37 Å, and the *X*(*i*) *vs.* delta(*d*) and delta(*p*) *vs.* delta(*a*) plots for all the nine compounds reveal in general a small extent of stretching in one structure compared to the other. *D* values of 0.09 and 0.06 are shortest for the molecular pair with highest 3D SC (BUVCUS/BUVDON) and 2D SC (BUVDON/BUTQEO) similarity, respectively, whereas *D* values of 0.37 and 0.31 are longest for molecular pair with lowest 3D SC (BUVDUT/BUTPUD) and 2D SC (BUVCOM/BUVCIG) similarity, respectively, as expected.

**Fig. 2:**
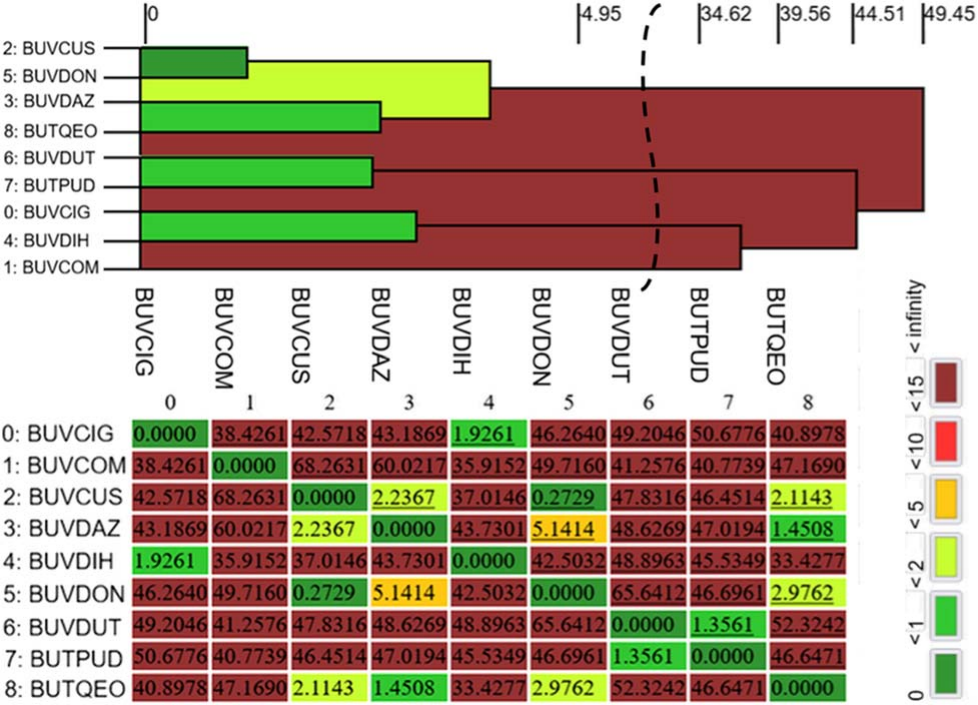
Dendrogram showing the packing similarity in the series of nine AOEB. The horizontal axis corresponds to the *PS*_ab_ value (similarity). Dark green indicates almost identical packing and dark red indicates dissimilar packing. Part of the dendrogram between 6.18 and 34.61 was removed to make the figure shorter; the vertical curved line indicates this deletion.

The packing similarity PS_ab_ was calculated using the CrystalCMP software.^19^ Molecular packing similarities and differences were analyzed through a packing similarity tree diagram, which facilitates a comprehensive evaluation of the relationships within a group of structures.^50^ The resulting dendrogram and its corresponding similarity matrix for the set of nine structures are presented in Fig. 2. The PS_ab_ values were extracted from this matrix, with comparisons performed based on the atomic kernel defined by the SMILES string: O(CC(O)C)C(O)C(C)C.

The packing similarity dendrogram allows for the identification of three distinct isostructural families.^51^ The highest degree of similarity is observed in the first family, comprising structures BUVCUS and BUVDON, which cluster at a PS_ab_ value of 0.2729 (indicated by a dark green link, 0 ≤ PS_ab_ ≤ 1). It is worth noting that for the pair BUVCUS and BUVDON, the *P*2_1_/*c* cell (BUVCUS) in the *P*2_1_/*n* setting (transformation: 100-1-0-10-1) becomes nearly identical to the *P*2_1_/*n* cell of BUVDON. This clear isostructurality, which is confirmed by the analyzed quantitative approach even when masked by different unit cell settings, serves as an additional proof of the robustness of the used framework.

A second family, representing moderate packing similarity, encompasses six additional structures (BUVDUT, BUVPUD, BUVDAZ, BUTQEO, BUVCIG, and BUVDIH) linked in the range 1 < PS_ab_ ≤ 2 (light green links). Within this group, three pairs exhibit PS_ab_ values in increasing order: BUVDUT/BUTPUD (1.3561) < BUVDAZ/BUTQEO (1.4508) < BUVCIG/BUVDIH (1.9261). Furthermore, a third family is identified in the range 2 < PS_ab_ ≤ 5 (greenish-yellow links), involving structures such as BUVCUS, BUVDON, BUVDAZ, and BUTQEO, with specific pair values of 2.1143 (BUVCUS/BUTQEO), 2.2367 (BUVCUS/BUVDAZ), and 2.9762 (BUVDON/BUTQEO).

The least close-packing relationship, within the range 5 < PS_ab_ ≤ 10 (orange link), is represented by the BUVDAZ/BUVDON pair with a PS_ab_ value of 5.1414. Furthermore, a group of three structures exhibits a highly pronounced packing dissimilarity (PS_ab_ > 15). This is specifically evident for BUVCOM/BUVDIH and BUVCOM/BUVCIG pairs, which show PS_ab_ values of 35.9152 and 38.4261, respectively, and are both characterized by dark red links. It is interesting to note that the four molecular pairs with the lowest PS_ab_ values (0.2729–1.9261) correspond to the same pairs where 3D structural similarity was identified through XPac calculations. Surprisingly, however, no direct correlation between the dissimilarity index (*x*) and the packing similarity (PS_ab_) values was observed.

All these results indicate that while previous studies on this molecular system relied on qualitative visual overlays to assess isostructurality, this work has provided a rigorous quantitative framework. By employing geometric descriptors, the isostructural relationships have been systematically categorized through supramolecular constructs and dendrogram analysis, ensuring a precise and reproducible identification of isostructural families that goes beyond simple graphical comparisons.

### 3.3 Non-covalent interactions and energy calculations

In order to evaluate the energetics associated with the primary intermolecular interactions in the crystal packing, calculations were performed using the CLP-PIXEL package^52^ and the Charger tool of MoProViewer.^53^ The resulting Pixel lattice energies and intermolecular interaction energies for selected molecular pairs are summarized in Tables 3 and 4, respectively. The lattice energy analysis reveals that the dispersion term (*E*_disp_) is the dominant contribution to the crystal stabilization for all nine compounds, as is typically observed for organic systems. The *E*_disp_ values fall within a narrow range of 143.2–164.0 kJ mol^−1^, accounting for an average of 73.8% of the total cohesive energy in the crystals.

**Table 3:** Lattice energies and their PIXEL components (kJ mol^−1^) for the AOEB series

Compound	*E*_coul_	*E*_pol_	*E*_disp_	*E*_rep_	*E*_tot_
BUVCUS	−41.1	−17.6	−162.7	86.9	−134.5
BUVDAZ	−39.5	−17.9	−164.0	90.1	−131.3
BUVDUT	−33.8	−14.8	−153.0	78.5	−123.1
BUVCIG	−37.3	−16.2	−143.2	82.5	−114.3
BUVDON	−34.4	−19.0	−152.3	82.1	−123.6
BUTQEO	−44.8	−18.0	−157.6	86.3	−134.0
BUTPUD	−33.5	−15.3	−157.4	80.2	−126.0
BUVDIH	−32.7	−14.9	−143.4	73.4	−117.5
BUVCOM	−43.6	−19.4	−159.3	87.7	−134.6

**Table 4:** PIXEL interaction energies partitioned into coulombic, polarization, dispersion, and repulsion contributions (kJ mol^−1^)

	Symmetry	Contacts^a^	Geom^b^	*D*_c_ c	*E*_coul_	*E*_pol_	*E*_disp_	*E*_rep_	*E*_tot_
BUVCIG	1 + *x*, *y*, *z*	C12B–H12D⋯O2A	2.498, 167	5.448	−17.1	−5.9	−46.0	30.3	−38.7
		C4B–H4BA⋯*C*g5	2.73, 4.03						
	−1 + *x*, 1 + *y*, *z*	C12A–H12B⋯O2B	2.348, 159	5.475	−15.1	−5.4	−41.8	25.2	−37.0
		C6Y–H6YA⋯*C*g13	2.94, 8.99						
	2 + *x*, − 1 + *y*, *z*	C18B–H18B⋯O2A	2.430, 142	10.925	−7.8	−3.0	−19.5	11.7	−18.5
	1 − *x*, 1 − *y*, −*z*	C18A–H18A⋯O3B	2.346, 141	9.650	−10.2	−4.5	−21.2	18.7	−17.2
BUVCOM	*x*, *y*, *z*	C12A-H12B⋯O2B	2.443, 163	5.208	−19.4	−7.4	−53.2	32.1	−47.6
	−1 + *x*, *y*, *z*	C12B–H12C⋯O2A	2.428, 174	5.388	−16.3	−5.9	−48.1	26.5	−43.7
		C4B–H4BA⋯Cg5	2.97, 8.54	10.264	−16.4	−7.5	−32.0	27.7	−28.2
	1 − *x*, − *y*, 1 − *z*	C18B–H18B⋯O3A	2.637, 129	10.264	−16.4	−7.5	−32.0	27.7	−28.2
		C17B–H17B⋯Cl1A	2.773, 171						
		C9X–H9XA⋯Cl1B	2.919, 125						
	−*x*, − *y*, 1 − *z*	Cg13⋯Cg13	3.870	10.986	−4.6	−2.2	−35.6	15.6	−26.8
	*x*, 1/2 − *y*, 1/2 + *z*	C3B–H3BB⋯O3B	2.574, 141	10.877	−5.5	−2.8	−17.2	8.8	−16.7
	*x*, 1/2 − *y*, − 1/2 + *z*	C18A-H18A⋯O2A	2.476, 157	8.312	−3.4	−2.4	−18.8	8.5	−16.2
	−1 + *x*, 1/2 − *y*, 1/2 + *z*	C3B–H3BA⋯Cl1A	2.949, 132	11.779	−2.5	−1.1	−11.1	5.8	−9.0
BUVCUS	−*x*, 1/2 + *y*, 3/2 − *z*	C9–H9A⋯O1	2.624, 145	5.316	−17.7	−7.8	−51.7	31.9	−45.2
	−*x*, − 1/2 + *y*, 3/2 − *z*	C7–H7A⋯O2	2.605, 137	5.316	−17.7	−7.8	−51.7	31.9	−45.2
	−*x*, 2 − *y*, 1 − *z*	C19–H19A⋯O3	2.489, 147	8.714	−9.3	−4.6	−24.6	15.3	−23.2
	1 + *x*, 1/2 − *y*, 1/2 + *z*	C6–H6A⋯Cl1	2.986, 136	14.078	−2.0	−0.7	−8.3	4.0	−7.1
BUVDAZ	1 − *x*, 1 − *y*, 1 − *z*	C12–H12A⋯Cg5	2.98, 17.68	7.219	−11.1	−5.5	−60.4	33.1	−43.9
	1 − *x*, 1/2 + *y*, 1/2 − *z*	C7–H7A⋯O2	2.661, 138	5.288	−15.7	−7.6	−47.7	29.1	−41.0
	1 − *x*, − 1/2 + *y*, 1/2 − *z*	C1–H1B⋯O1	2.629, 148	5.288	−15.7	−7.6	−47.7	29.1	−41.0
	1 − *x*, − *y*, 1 − *z*	C15–H15A⋯O3	2.478, 139	8.709	−11.4	−5.1	−30.8	18.4	−28.8
	1 + *x*, 1/2 − *y*, − 1/2 + *z*	C10–H10A⋯Cl1	2.954, 142	16.318	−2.1	−0.9	−9.6	5.0	−7.7
BUVDIH	*x*, 1 + *y*, *z*	C12B–H12D⋯O2A	2.440, 174	5.150	−15.4	−5.8	−47.5	25.8	−42.9
	*x*, *y*, *z*	C4A-H4AA⋯Cg13	2.99, 2.84	5.303	−14.2	−5.5	−45.4	24.6	−40.5
		C12A-H12A⋯O2A	2.430, 168						
	1 − *x*, 1 − *y*, − *z*	C18A-H18A⋯O3B	2.708, 149	10.376	−1.2	−1.4	−26.4	8.5	−20.6
	−1 + *x*, *y*, *z*	C1B–H1BA⋯*C*g13	2.96, 6.00	9.721	−4.1	−2.6	−25.8	15.0	−17.6
	−1 + *x*, 1 + *y*, *z*	C10B–H10F⋯O3A	2.703, 139	8.404	−3.6	−2.3	−22.2	11.3	−16.8
	1 + *x*, *y*, *z*	C18B–H18B⋯O2A	2.403, 165	10.780	−6.1	−2.6	−16.0	9.5	−15.1
BUVDON	3/2 − *x*, 1/2 + *y*, 1/2 − *z*	C1–H1B⋯O1	2.637, 145	5.188	−16.2	−8.1	−51.1	31.6	−43.8
	3/2 − *x*, − 1/2 + *y*, 1/2 − *z*	C7–H7B⋯O2	2.606, 137	5.188	−16.2	−8.1	−51.1	31.6	−43.8
	1 − *x*, 2 − *y*, −*z*	C12–H12B⋯Cg5	2.97, 20.55	6.500	−10.1	−5.5	−53.8	29.3	−40.2
	1 − *x*, 3 − *y*, −*z*	C19A-H19A⋯O3	2.528, 138	8.500	−7.6	−4.5	−23.7	14.4	−21.5
BUVDUT	1 − *x*, 1 − *y*, −*z*	C7–H7B⋯Cg5	2.85, 6.67	5.744	−13.9	−5.3	−59.8	34.6	−44.4
	2 − *x*, 1 − *y*, −*z*	C18–H18A⋯O2	2.448, 161	9.206	−14.2	−6.1	−34.8	22.2	−32.9
	2 − *x*, − *y*, −*z*	C20–H20C⋯O2	2.470, 145	10.629	−10.0	−4.3	−31.6	16.0	−30.0
		Cg5⋯Cg5	3.878						
BUTPUD	−*x*, 2 − *y*, −*z*	C7–H7A⋯Cg5	2.82, 7.80	5.538	−14.0	−5.7	−60.8	35.4	−45.1
	1 − *x*, 2 − *y*, −*z*	C20–H20B⋯O2	2.618, 135	9.432	−10.8	−5.6	−36.6	19.1	−33.9
	1 − *x*, 3 − *y*, −*z*	C20–H20C⋯O3	2.612, 162	11.095	−5.5	−3.7	−32.7	13.7	−28.8
	−*x*, 3 − *y*, −*z*	C15–H15A⋯O3	2.614, 126	8.050	−1.9	−3.4	−22.4	10.2	−17.5
BUTQEO	1 − *x*, − 1/2 + *y*, 1/2 − *z*	C1A-H2⋯O1A	2.646, 150	5.292	−15.6	−6.9	−47.1	28.2	−41.4
	1 − *x*, 1/2 + *y*, 1/2 − *z*	C9A-H13⋯O2A	2.685, 136	5.292	−15.6	−6.9	−47.1	28.2	−41.4
	1 − *x*, 2 − *y*, −*z*	C7–H12A⋯Cg5	2.92, 14.16	6.829	−6.7	−5.2	−53.1	24.9	−40.1
	1 − *x*, 1 − *y*, −*z*	C19A-H19A⋯O3A	2.488, 132	8.404	−17.6	−6.3	−32.6	21.8	−34.7
		C6A-H9⋯O5A	2.681, 134						
	2 − *x*, 2 − *y*, −*z*	C16A-H16A⋯O4A	2.383, 152	16.353	−13.3	−3.3	−10.3	11.6	−15.3

a Cg5 is the centroid of ring C14A–C19A for BUVCIG, BUVCOM and BUTQEO; Cg13 is the centroid of ring C14B–C19B for BUVCIG and BUVCOM; Cg5 is the centroid of ring C14–C19 for BUVDUT and BUTPUD.

b H⋯O/Cl distance and C–H⋯O/Cl angle for H-bonds; H⋯Cg distance, *γ* (angle between Cg–H vector and ring J normal) for C–H⋯Cg.

c Inter-centroid distance.

A common structural feature in the packing of all nine AOEB is the formation of intermolecular non-classical C–H⋯O hydrogen bonds and weak C–H⋯π contacts (with the exception of BUVCUS), the latter linking the adamantane group to the phenyl ring. Additionally, H⋯Cl hydrogen bonds are present in the three chloro derivatives (BUVCOM, BUVCUS, and BUVDAZ), while the crystal packing is further consolidated by π⋯π interactions in BUVCOM and BUVDUT. The short H⋯O distances (2.346–2.708 Å), which remain below the sum of the van der Waals radii (2.72 Å), along with C–H⋯O bond angles in the 126°–174° range, confirm the presence of these H-bonds. In some dimers, these interactions appear as the primary attractive force, yielding intermolecular energies in the highest range of 41.4–45.2 kJ mol^−1^, consistent with the shortest inter-centroid distances (5.150–5.316 Å). As expected, these energies decrease to 15.1–33.9 kJ mol^−1^ as the inter-centroid distances increase to 8.050–16.353 Å.

The solid-state structures of all nine compounds, with the exception of BUVCUS, are further stabilized by intermolecular C–H⋯π contacts to the phenyl ring. These interactions were identified using the geometric criteria implemented in PLATON for X–H⋯Cg(π-ring) analysis: an H⋯Cg distance <3.0 Å and an angle between the Cg–H vector and the ring normal <40.0°. The geometric parameters for these contacts are summarized in Table 4 (column 4). Notably, C–H⋯π contacts with inter-centroid distances between 5.303 and 7.219 Å constitute the sole attractive force between molecular pairs in most compounds (excepting BUVCIG and BUVCOM). The associated intermolecular energies, ranging from −40.1 to −45.1 kJ mol^−1^, indicate that these contacts serve as highly stabilizing structural motifs within the supramolecular environment.

In an effort to estimate the energetic contribution of individual contacts to the total interaction energy in dimers involving multiple contact types, total electrostatic interaction energies *E*^elec^_tot_ were calculated using the Charger tool (Table S1). The analysis focused on donors (C–H groups) and acceptors (O, Cl, and the aromatic phenyl ring π-systems). The results show that individual contact energies fall within a narrow range of −2.63 to −8.22 kJ mol^−1^, suggesting that the supramolecular stability arises from a cooperative sum of comparable contributions rather than a single dominant force. For instance, in BUVCOM (Dimer 1) and BUVDUT, the C–H⋯π and C–H⋯O interactions provide remarkably similar percentages of the total electrostatic energy (ranging from 12.8% to 17.5%).

A similar balance is observed in BUVCIG (Dimer 2), where the C–H⋯π and C–H⋯O contacts contribute 15.3% and 20.2%, respectively. This stands in contrast to BUVCIG (Dimer 1), where the C–H⋯π contact (22.1%) significantly outweighs the C–H⋯O bond (9.9%). Interestingly, in BUVCOM (Dimer 2), the contribution of the C–H⋯Cl contacts (19.7% and 15.0%) is notably more significant than that of the C–H⋯O interaction (10.1%). This preference likely stems from the differences in polarizability and electronegativity between chlorine and oxygen atoms in this specific chemical environment. Overall, these results demonstrate that, with few exceptions, the various non-covalent contacts involved in dimer formation provide comparable contributions to the total energetic stability of the crystalline architecture described for these systems.

### 3.4 Hirshfeld surface analysis

**Fig. 3:**
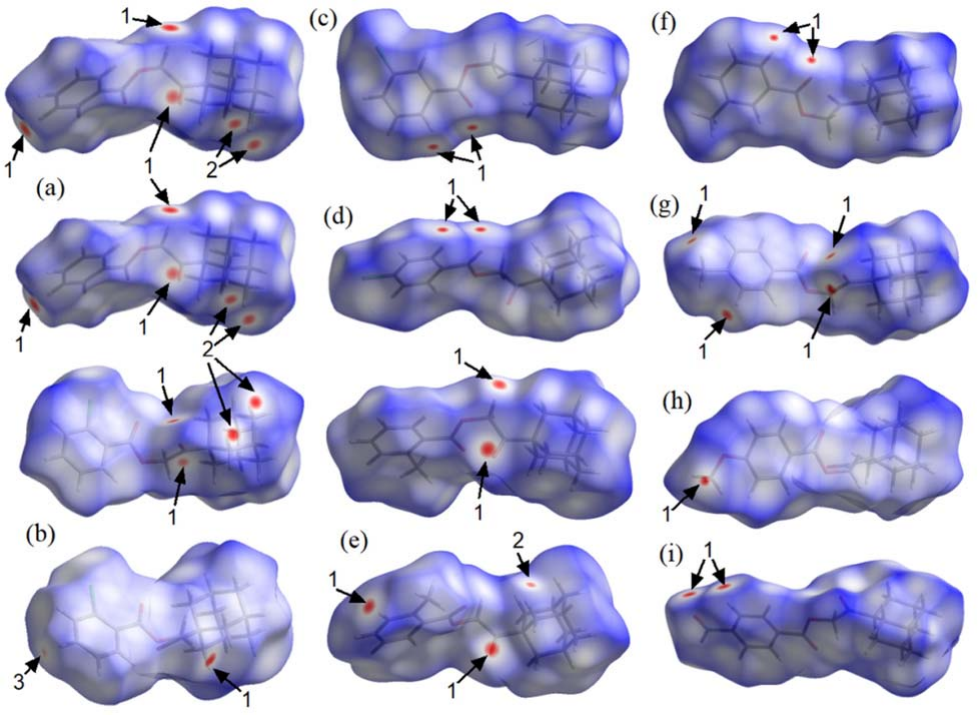
Hirshfeld surfaces mapped over *d*_norm_ showing intermolecular (1) O⋯H/H⋯O, (2) H⋯H and (3) Cl⋯H/H⋯Cl contacts for (a) BUVCIG, (b) BUVCOM, (c) BUVCUS, (d) BUVDAZ, (e) BUVDIH, (f) BUVDON, (g) BUVDUT, (h) BUTPUD, and (i) BUTQEO.

Hirshfeld surface (HS) analysis was carried out to evaluate the intermolecular interactions and their relative strengths in maintaining the crystalline lattice.^54,55^ HS mapped over *d*_norm_ (Fig. 3) reveal localized red spots associated with C–H⋯O hydrogen bonds as a universal feature. While H⋯H contacts are abundant, they only generate significant dnorm features in compounds with *Z*′ = 2. Notably, C–H⋯Cl interactions are only visually prominent in the *ortho*-chloro derivative, whereas in the *meta* and *para* analogues, the distances exceed the van der Waals radii, confirming that only weak, localized contacts govern the crystal packing.

The 2D fingerprint plots (Fig. S11) show that H⋯H interactions (44.4–69.8%) and O⋯H/H⋯O contacts (15.2–33.4%) dominate the surfaces. C⋯H/H⋯C interactions, representing C–H⋯π contacts, constitute the third-largest contribution (10.2–16.9%). Although the directionality of these interactions is still debated,^56^ they are generally directed toward the aromatic ring centroid.^57^ Interestingly, even when formal C–H⋯*Cg* contacts are not detected by geometric criteria, HS analysis captures significant local H⋯C interactions that contribute to the overall stabilization of the crystal packing.

Shape-index analysis further clarifies the role of π-stacking interactions. The evidence of C–H⋯π contacts is particularly clear in the shape-index surface (Fig. S12). A “bow-tie” pattern, indicative of π⋯π stacking,^10^ with inter-centroid distances < 3.8 Å, is only observed for BUVCOM and BUVDUT (Fig. S13). This observation is in full agreement with the geometric parameters calculated by PLATON, which consistently indicate that the aromatic rings are positioned in a geometry that precludes effective face-to-face or offset stacking interactions.

Finally, enrichment ratios (*E*_XY_, Table S2) highlight contact propensities.^58^ C⋯C interactions are highly enriched in BUVCOM and BUVDUT (*E*_CC_ ≈ 1.7), correlating with π⋯π stacking. For all compounds, O⋯H/H⋯O contacts are favored (*E*_OH_ > 1), with the ketone oxygen usually acting as the acceptor, except in BUVDIH and BUTPUD. In contrast, C⋯H/H⋯H contacts are unprivileged, showing that assembly is driven by hydrogen bonding and aromatic stacking.

### 3.5 DFT study of adamantane⋯benzoate interactions

**Fig. 4:**
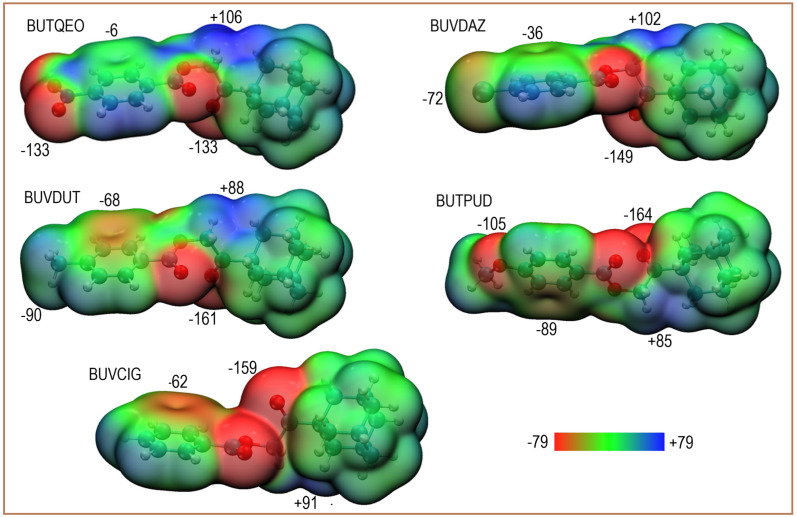
Molecular electrostatic potential (MEP) surfaces for (a) BUTQEO, (b) BUVDAZ, (c) BUVDUT, (d) BUTPUT, and (e) BUVCIG mapped onto the electron density isosurface (0.001 a.u.). Local MEP values are shown. The color scale ranges from −78.8 (red) to +78.8 kJ mol^−1^ (blue).

Before analyzing intermolecular interactions, the characterization of the monomeric electrostatic landscape by MEP is essential. A selection of five *para*-substituted derivatives (Fig. 4), ranging from electron-withdrawing –NO_2_ to electron-donating –OCH_3_, was chosen to evaluate how their electronic nature modulates the aromatic system and the adamantane cage (Table S3).

The MEP surfaces reveal a topological “well” at the ring center where the potential becomes progressively less negative from –OCH_3_ (−89 kJ mol^−1^) to –NO_2_ (−6 kJ mol^−1^), correlating with the substituent's electronic character. The carbonyl oxygens act as primary nucleophilic sites, with potentials tuned by the substituents (−164 kJ mol^−1^ for –OCH_3_*vs.* −133 kJ mol^−1^ for –NO_2_), while the central ester oxygen shows a weaker character due to resonance. Notably, in the chloro derivative the potential minimum migrates towards the oxygen adjacent to the phenyl ring. Meanwhile, the methylene bridge consistently maintains a stable electrophilic site (∼+90 kJ mol^−1^).

**Fig. 5:**
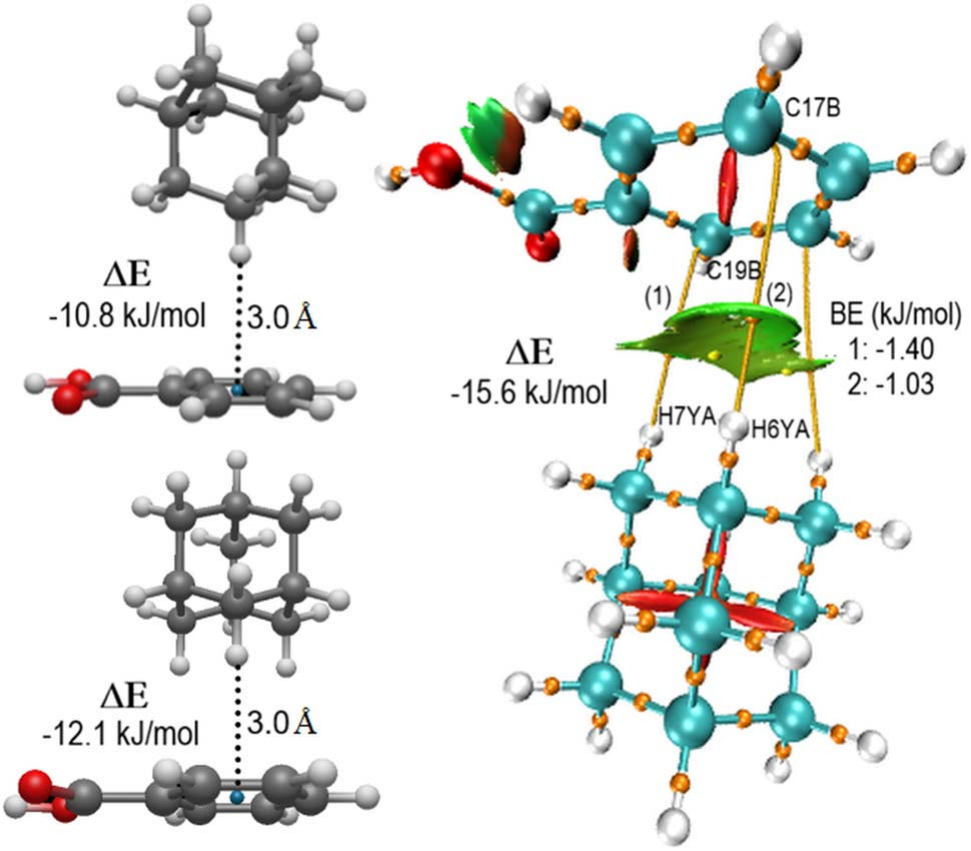
Theoretical models for methylenic and methinic dimer motifs (left) and QTAIM/NCI-plot analysis for the BUVCIG compound (right). Dimerization energies (Δ*E*) and binding energies (BE) are given in kJ mol^−1^. Atomic labels highlight the C and H atoms involved in the stabilized contacts.

Topological properties at Bond Critical Points (BCPs) were evaluated using QTAIM and NCI-Plot^10^ for all the nine compounds to characterize C–H⋯π contacts (see Table S4). QTAIM-NCI diagrams for BUVCIG and the two vacuum models are illustrated in Fig. 5. These interactions were defined by a cutoff H⋯C distance of 3.2 Å.^59^ H-bond binding and dimerization energies for the geometrically strongest five derivatives are shown in Table S5. The interaction energies were calculated through Single Point Energy (SP) calculations using the atomic coordinates obtained directly from the single-crystal X-ray diffraction of the nine structures. This approach was chosen to preserve the fidelity of the intermolecular interactions as they exist within the crystalline packing. Given the high resolution of the experimental data, a gas-phase geometry optimization could induce artificial relaxations that do not represent the actual chemical environment of the crystal. Residual *R* factors and GoF values are shown in Table S6.

Idealized interaction models (methylene and methine dimers) were constructed using adamantane and benzoate geometries optimized with the Universal Force Field (UFF) in Avogadro. These models were used to calibrate the C–H⋯π interaction energies in a simplified environment. Cartesian coordinates of adamantane and benzoate fragments are listed in Table S7. SP energy data from the output files of the nine structures and the two optimized Models are given in Table S8. The methylene (1, 2.953 Å) and methine (2, 3.063 Å) contacts exhibit BE values of −1.40 and −1.03 kJ mol^−1^, respectively, which correlate well with their respective interatomic distances.

As shown in Table S5, the dimerization energy (Δ*E*) for the BUVCIG experimental dimer (−15.56 kJ mol^−1^) is significantly higher than those calculated for the methylene (−12.1 kJ mol^−1^) and methine (−10.8 kJ mol^−1^) vacuum models, suggesting that lattice-induced compression enhances the interaction beyond the baseline stability of isolated fragments. The binding energies (BE) also confirm the superior H-bond donor capacity of methylene groups (average BE = −1.51 kJ mol^−1^) over methine groups (average BE = −1.08 kJ mol^−1^) in the formation of C–H⋯π interactions. Furhermore, the BEs for substituted derivatives (−17.78 to −20.00 kJ mol^−1^) show even greater stabilization, confirming the role of substituent effects in tuning these contacts.

The electronic origin of the assemblies was further investigated through Natural Bond Orbital (NBO) analysis (Table S9). Results for BUVCIG and the vacuum models reveal a significant charge transfer from the aromatic π-system to the σ*(C–H) antibonding orbitals of the adamantyl group. This π → σ* stabilization is evidenced by higher occupancies in methylene orbitals (0.015–0.017 *e*^−^) compared to methinic ones (0.012–0.014 *e*^−^), confirming the greater electrophilicity of the former.

Notably, BUVCIG exhibits the highest occupancy (0.017 *e*^−^) and stabilization energies (*E*^(2)^ = 7.70 kJ mol^−1^) for methylene contacts, correlating with its more negative dimerization energy (Δ*E*) relative to the methinic site (5.06 kJ mol^−1^). These values represent an enhancement over the vacuum models (*E*^(2)^ = 6.53 and 4.10 kJ mol^−1^, respectively), confirming that these C–H⋯π contacts are genuine hyperconjugative stabilizations that dictate the supramolecular architecture in this system, consistent with the identified topological BCPs. These results align with the trend reported for the adamantane-benzene models,^6^ where methylene groups exhibit stronger interactions than methine ones.

### 3.6 SwissADME predictions

Pharmacokinetic and medicinal chemistry profiles for all derivatives, obtained *via* the SwissADME tool,^60^ are summarized in Tables S10–S18. The Bioavailability Radar confirms the druglikeness for the entire series. Favorable lipophilicity is observed, with *i* Log *P* and *X* LOG *P*3 values ranging from 3.03 to 3.47 and 3.94 to 4.77, respectively. The moderate water solubility suggests a balanced profile between membrane permeability and aqueous solubility. Notably, the Topological Polar Surface Area (TPSA) is identical for all compounds (43.37 Å^2^), falling well within the thresholds for high intestinal absorption (TPSA < 140 Å^2^) and potential blood–brain barrier penetration (TPSA < 60 Å^2^). The medicinal chemistry profile reveals high structural quality with zero PAINS^61^ or Brenk alerts^62^ (except for BUTQEO). Despite a leadlikeness violation due to *X* LOG *P*3, the series shows moderate synthetic accessibility^63^ (4.45–4.70), confirming the pharmacological potential of these adamantane-benzoate hybrids.

## 4 Conclusions

In summary, this work provides a comprehensive quantitative characterization of the structural and electronic features of nine 2-(adamantan-1-yl)-2-oxoethyl benzoates. Crystal packing is governed by moderate C–H⋯O and weak π-mediated interactions. C–H⋯π contacts were identified in all derivatives except BUVCUS, while π⋯π stacking stabilizes only BUVCOM and BUVDUT systems. Calculation of dissimilarity indices and packing similarity dendrograms showed 3D and 2D structural relationships, identifying three distinct isostructural families.

PIXEL energy analysis calculations confirmed the dominance of C–H⋯O interactions. The results were further supported by Hirshfeld surface analysis and enrichment ratios (*E*_XY_), highlighting O⋯H, C⋯C, and C–H as the most favored contacts. DFT calculations (MEP, AIM, NCI-Plot, NBO) provided the electronic rationale for these observations, unraveling the distinct contributions of methylene and methine protons of the adamantane framework. Finally, *in silico* predictions confirmed that all nine compounds strictly adhere to druglikeness rules, with promising gastrointestinal absorption and blood–brain barrier penetration. Hence, this study serves as a robust reference for the future design of adamantane-based scaffolds in medicinal chemistry.

## Conflicts of interest

There are no conflicts of interest to declare.

## Supplementary Material

RA-016-D6RA01672C-s001

## Data Availability

The supporting data for this article, including computational results and analysis plots, have been included as part of the supplementary information (SI). Supplementary information: This study was carried out using publicly available crystallographic data retrieved from the Cambridge Crystallographic Data Centre (CCDC) for the following refcodes: BUVCIG, BUVCOM, BUVCUS, BUVDAZ, BUVDIH, BUVDON, BUVDUT, BUTPUD and BUTQEO. No new crystallographic data were generated during this study. Packing similarity figures; Hirshfeld surface analysis and DFT interaction energy data (tables and figures); and SwissADME prediction tables. See DOI: https://doi.org/10.1039/d6ra01672c.
