# Supplementary material for: Isostructurality and energetics of competitive C–H⋯π interactions in adamantane-benzoate hybrids: a computational ADME study
Source: RSC Adv. 2026 Jun 2;16(32):29280–90. doi: 10.1039/d6ra01672c (PMC13231375; doi:10.1039/d6ra01672c)
Supplement: RA-016-D6RA01672C-s001 [file RA-016-D6RA01672C-s001.pdf]

Supplementary Information (SI) for RSC Advances.
This journal is © The Royal Society of Chemistry 2026

RSC Advances

Supplementary Information

Isostructurality and Energetics of Competitive C–H $\cdots\pi$ Interactions in Adamantane-Benzoate Hybrids: A Computational ADME Study†

Hiram Pérez[a,*]

[a]Universidad de La Habana, Facultad de Química.
Zapata s/n, e/ G y Carlitos Aguirre, Vedado, Habana 10400, Cuba.

*Correspondence Author: E-mail: hiramperezperez49@gmail.com

List of Figures

pag.

S1 XPac results for BUVCIG–BUVDIH pair. S2
S2 XPac results for BUVDAZ–BUTQEO pair. S2
S3 XPac results for BUVDUT–BUTPUD pair. S3
S4 XPac results for BUVCUS–BUVDON pair. S3
S5 XPac results for BUVCIG–BUVCOM pair. S4
S6 XPac results for BUVCOM–BUVDIH pair. S4
S7 XPac results for BUVDAZ–BUVDON pair. S5
S8 XPac results for BUVDON–BUTQEO pair. S5
S9 XPac results for BUVCUS–BUVDAZ pair. S6
S10 XPac results for BUVCUS–BUTQEO pair. S6
S11 Fingerprint plots showing the percentage contributions for individual atom–atom contacts. S7
S12 View of the three-dimensional Hirshfeld surfaces mapped over shape-index for visualizing intermolecular C-H$\cdots\pi$ interactions. S8
S13 Hirshfeld surfaces of BUVCOM (left) and BUVDUT (right) mapped over shape-index showing the 'bow-tie' patterns of touching red and blue triangles depicting $\pi\cdots\pi$ interaction. S8

List of Tables

pag.

S1 Total electrostatic energies $E^{\text{elec}}_{\text{tot}}$ (kJ/mol)^a for dimers involving different types of contacts. S9
S2 Enrichment ratios E_{XY} (reciprocal contacts merged) of intermolecular interactions.[a] S10
S3 Calculated electrostatic potential (V_s) (in kJ/mol) at the surface (isosurface 0.001 a.u) of *para*-substituted AOEB derivatives. S11
S4 Topological properties (in a.u.) at the Bond Critical Point CP (3,−1) of selected intermolecular H$\cdots$C interactions^a S11
S5 QTAIM dimerization energies (ΔE), H$\cdots$C distances, and H-bond binding energy (BE) involving methylene and methine H-atoms for AOEB with C–H$\cdots\pi$ interactions (kJ/mol, Å). S12
S6 R factors and GoF values for five AOEB crystal structures S12
S7 Calculated energies and BSSE correction for five AOEB crystal structures S13
S8 UFF optimized cartesian coordinates (Å) for Adamantane and Benzoic acid fragments S13
S9 Selected NBO parameters for the C–H$\cdots\pi$ interactions in the experimental (BUVCIG) and idealized (Methylenic and Methinic) models. S14
S10 BOILED-Egg plot and SwissADME properties for BUVCIG S15
S11 BOILED-Egg plot and SwissADME properties for BUVCOM S16
S12 BOILED-Egg plot and SwissADME properties for BUVCUS S17
S13 BOILED-Egg plot and SwissADME properties for BUVDAZ S18
S14 BOILED-Egg plot and SwissADME properties for BUVDIH S19
S15 BOILED-Egg plot and SwissADME properties for BUVDON S20
S16 BOILED-Egg plot and SwissADME properties for BUVDUT S21
S17 BOILED-Egg plot and SwissADME properties for BUTPUD S22
S18 BOILED-Egg plot and SwissADME properties for BUTQEO S23

Figures

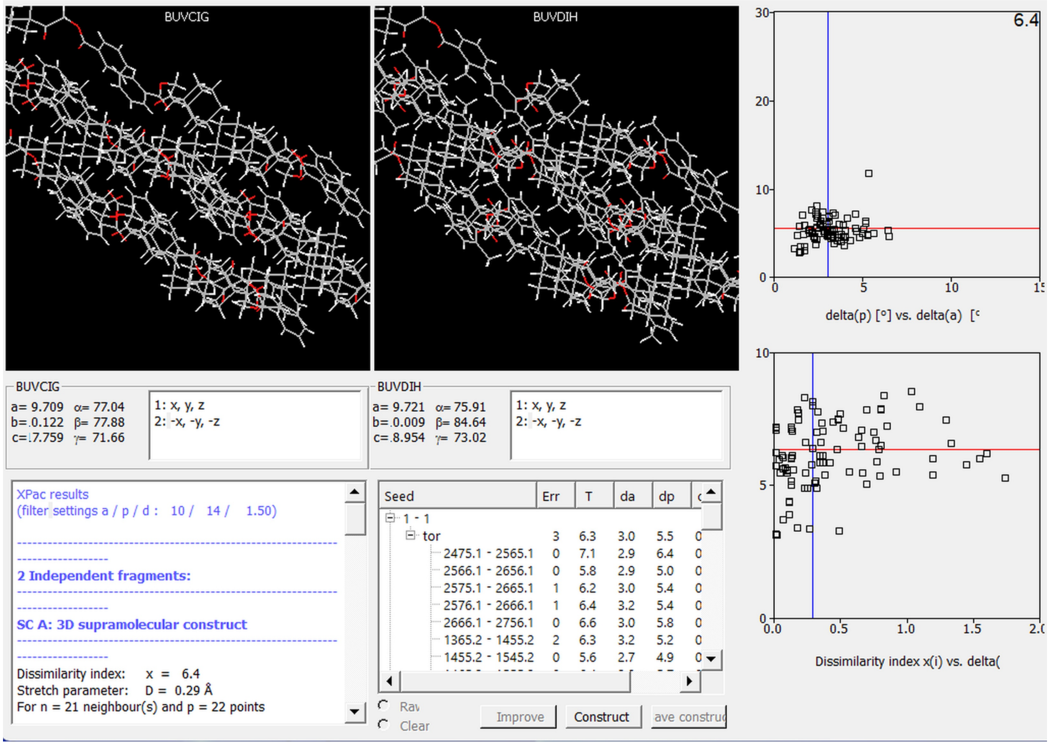

Figure S1. XPac results for BUVCIG–BUVDIH pair.

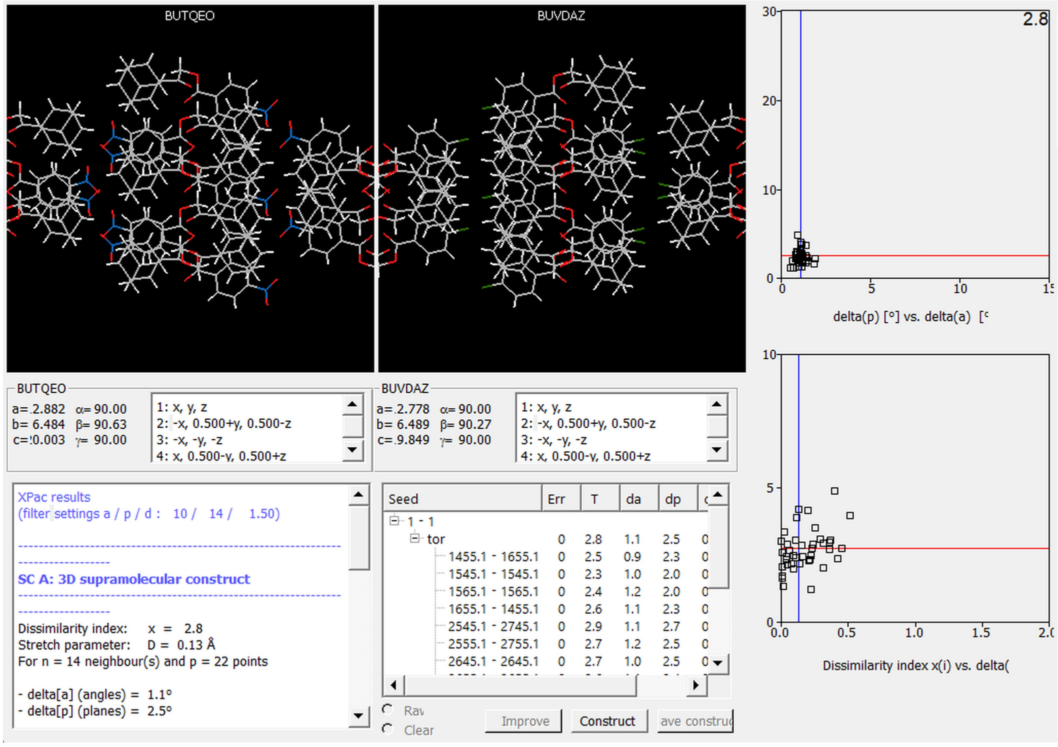

Figure S2. XPac results for BUVDAZ–BUTQEO pair.

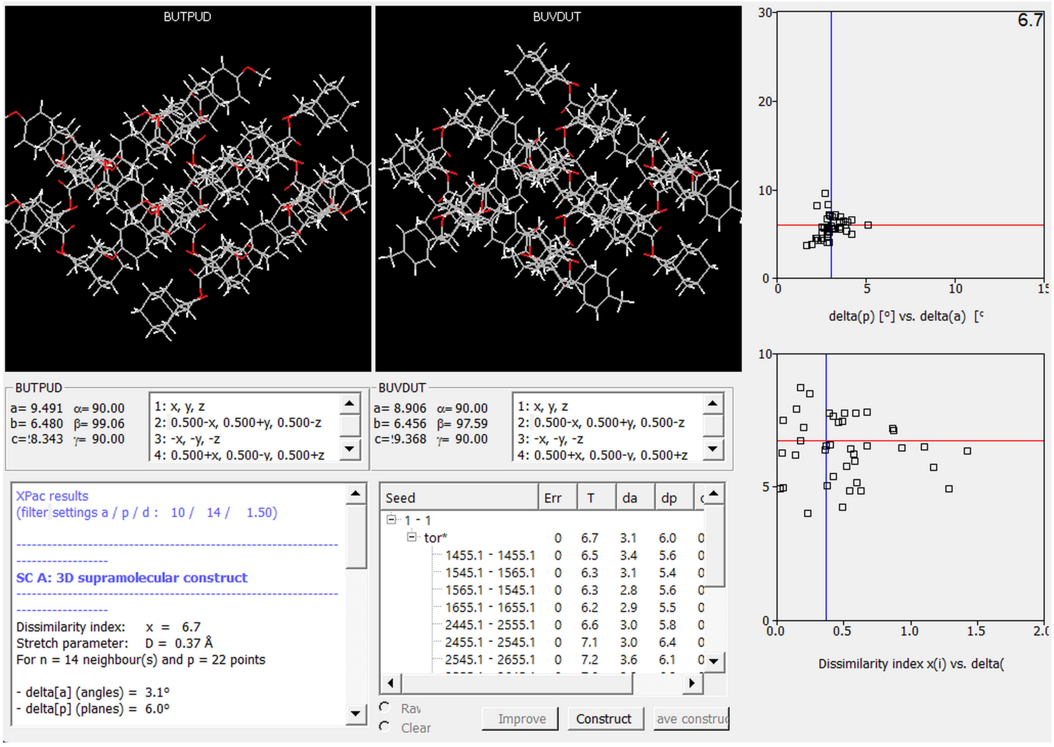

Figure S3. XPac results for BUVDUT–BUTPUD pair.

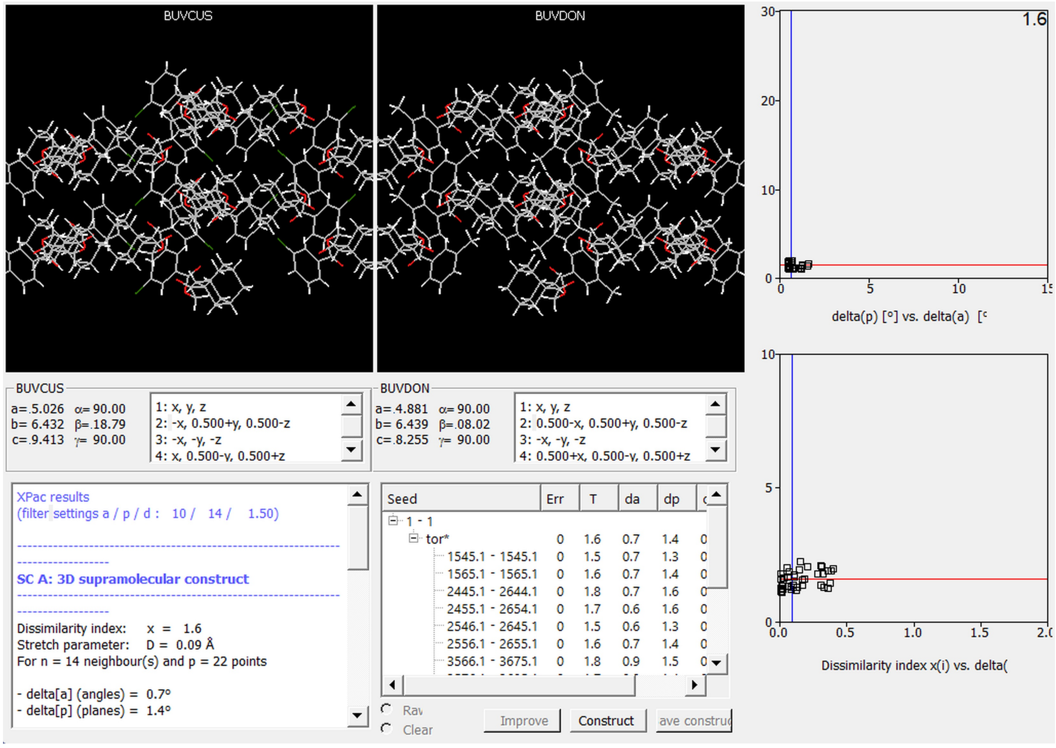

Figure S4. XPac results for BUVCUS–BUVDON pair.

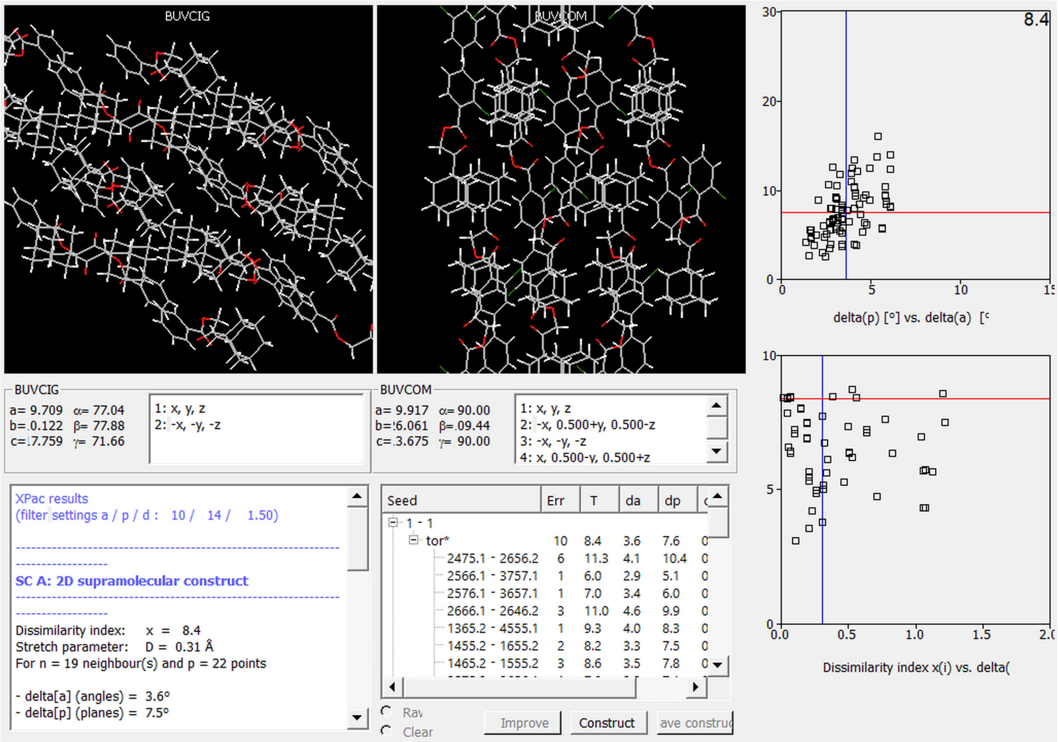

Figure S5. XPac results for BUVCIG–BUVCOM pair.

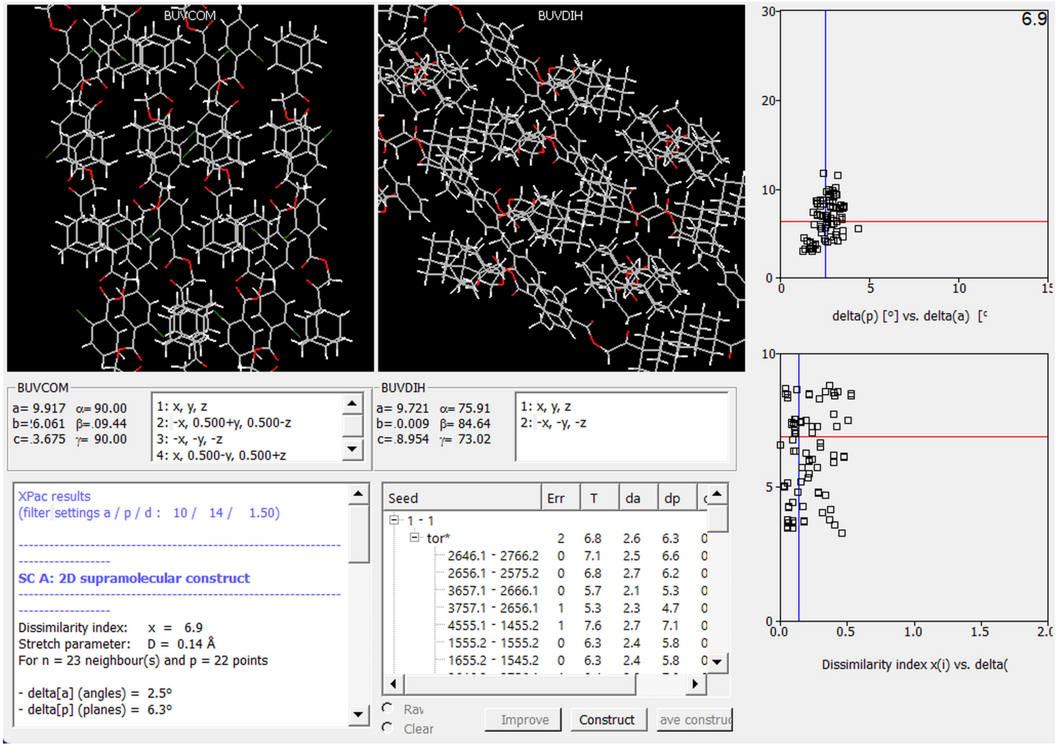

Figure S6. XPac results for BUVCOM–BUVDIH pair.

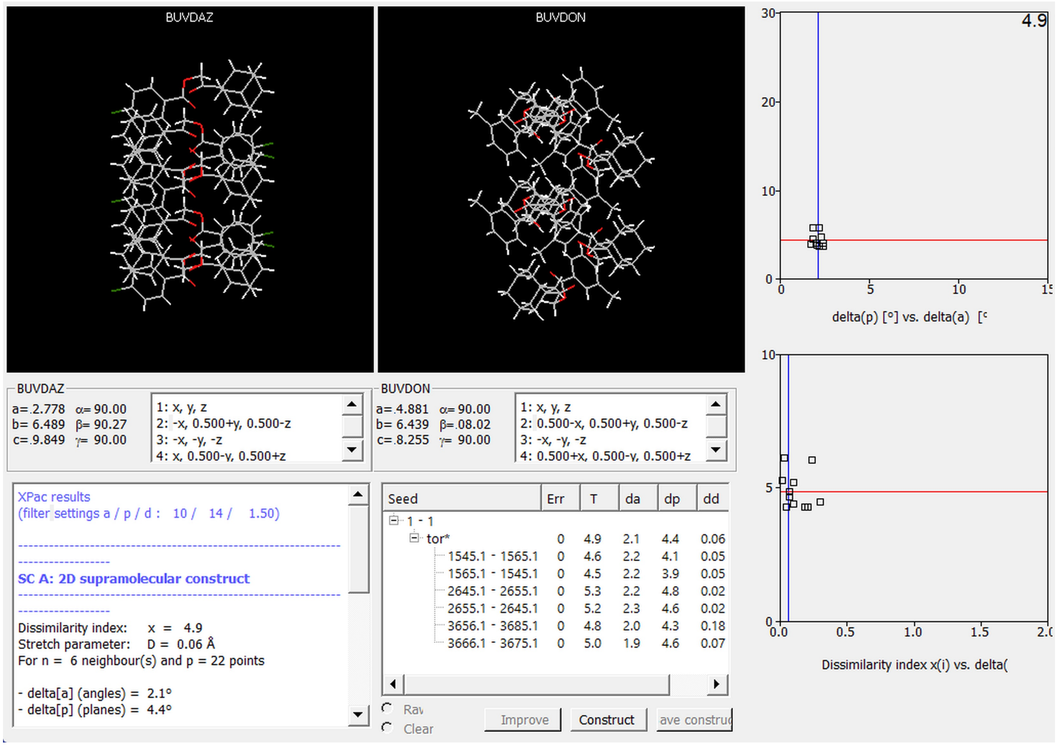

Figure S7. XPac results for BUVDAZ–BUVDON pair.

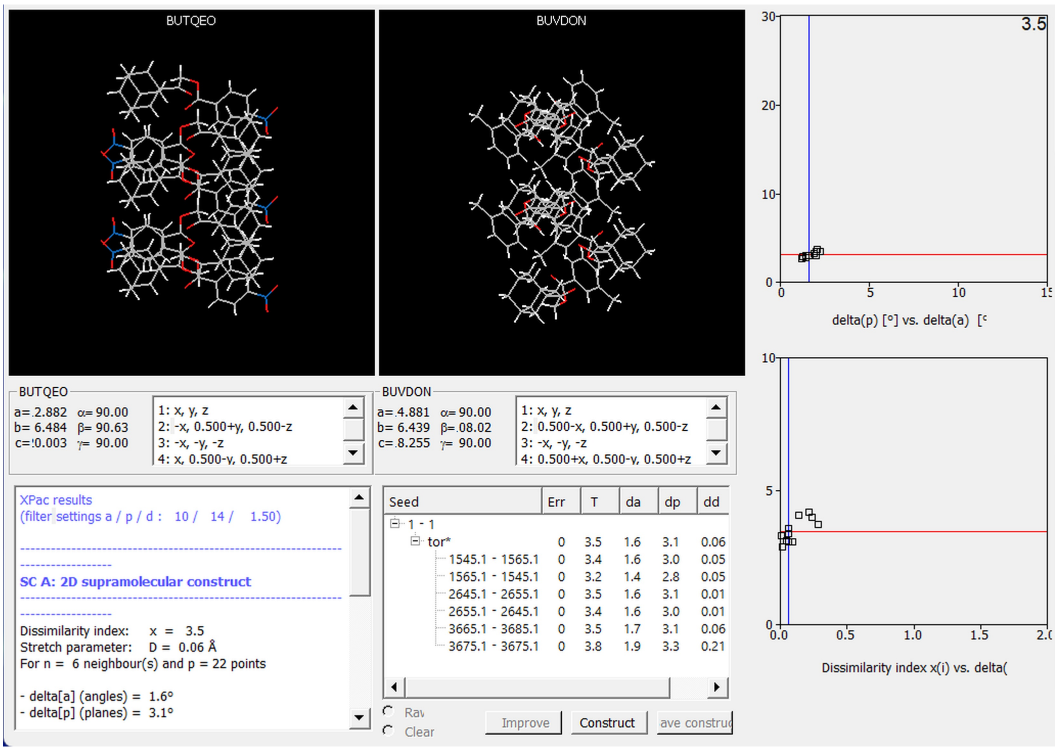

Figure S8. XPac results for BUVDON–BUTQEO pair.

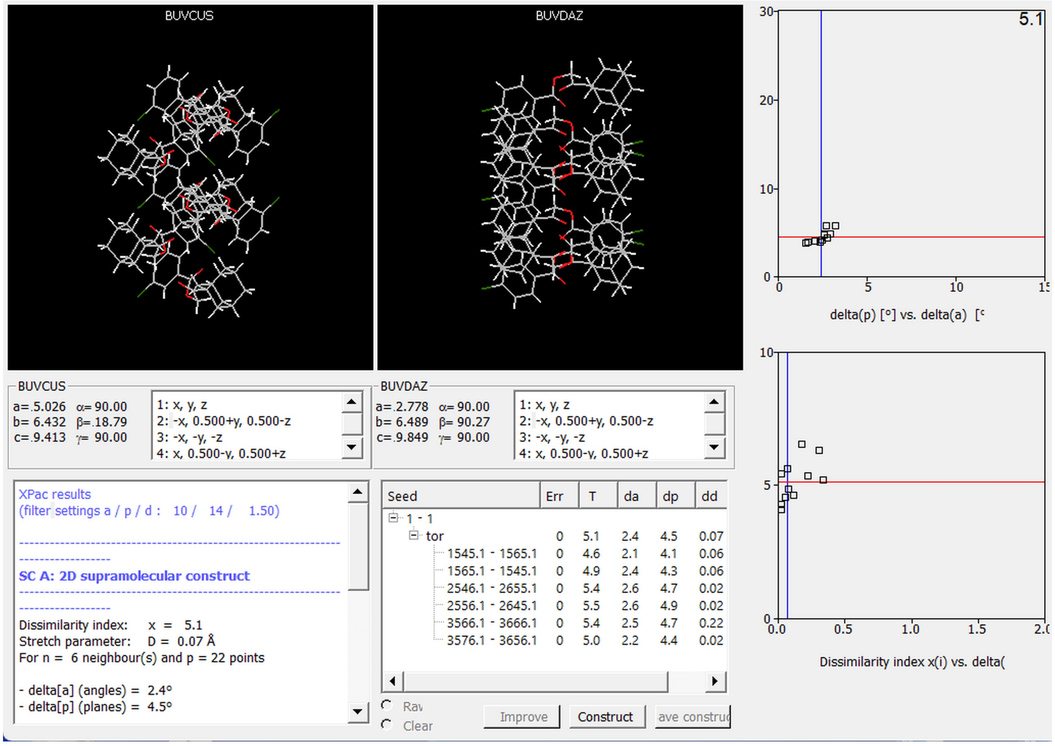

Figure S9. XPac results for BUVCUS–BUVDAZ pair.

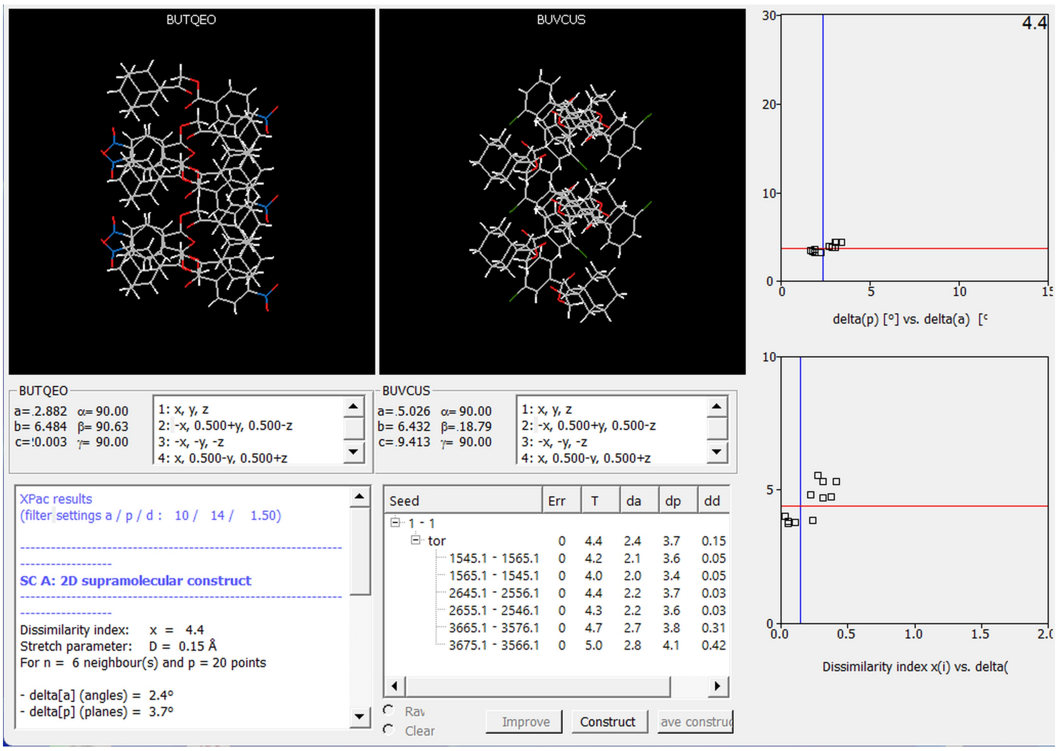

Figure S10. XPac results for BUVCUS–BUTQEO pair.

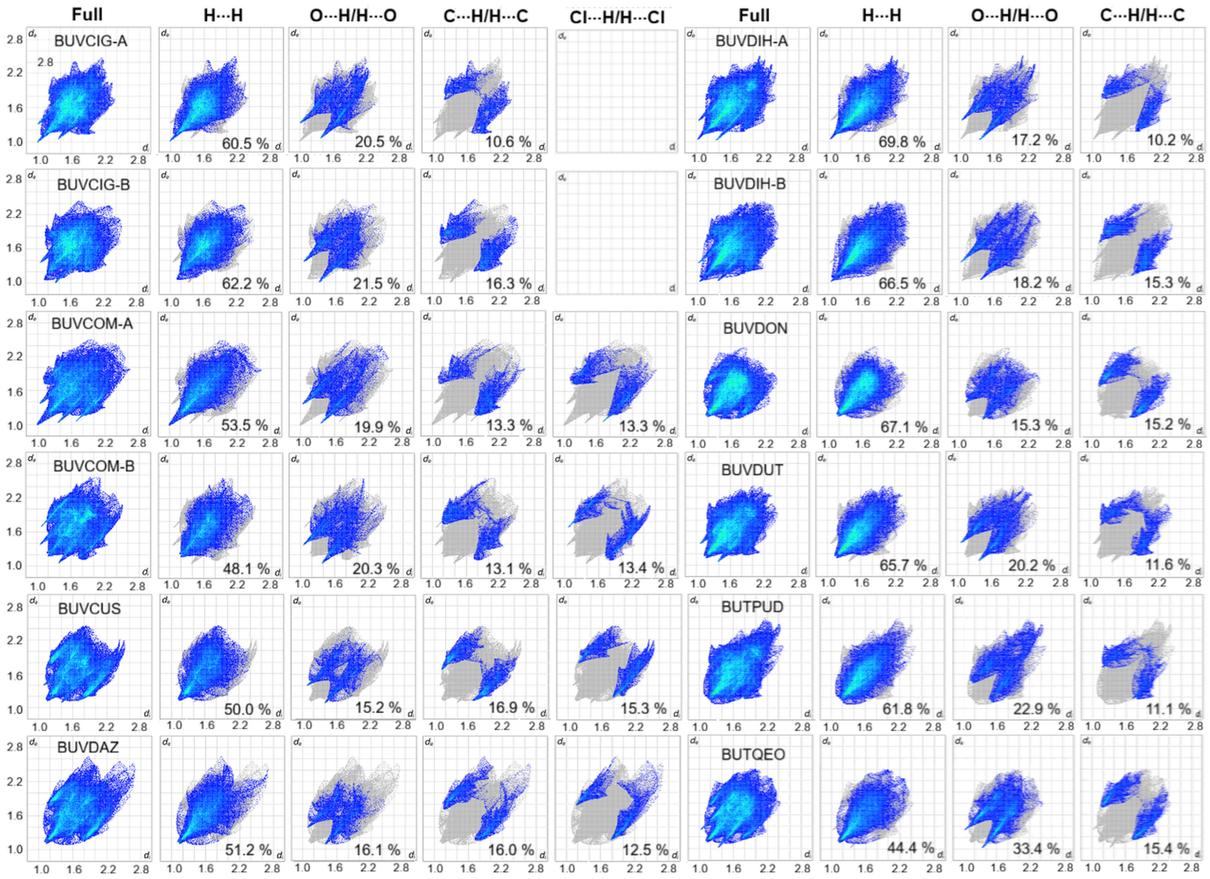

Figure S11. Fingerprint plots showing the percentage contributions for individual atom–atom contacts.

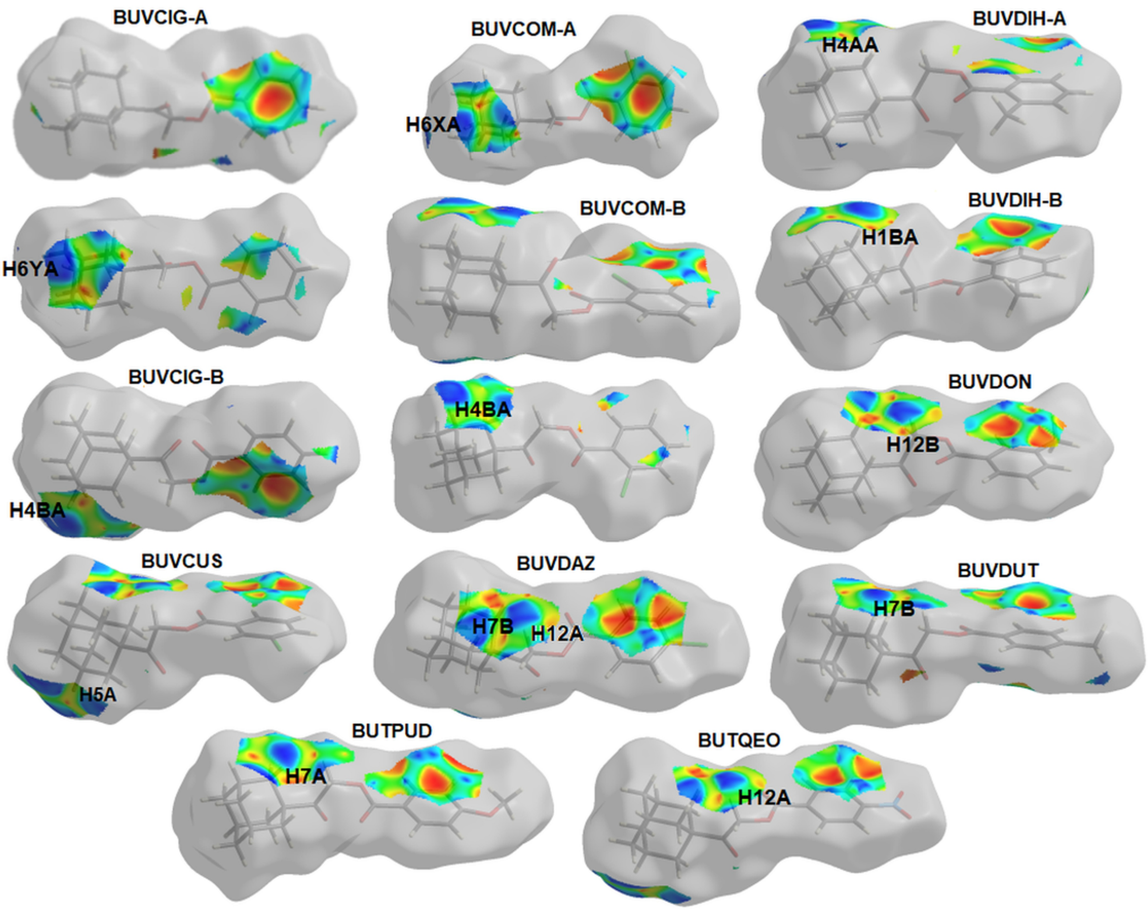

Figure S12. View of the three-dimensional Hirshfeld surfaces mapped over shape-index for visualizing intermolecular C-H$\cdots\pi$ interactions.

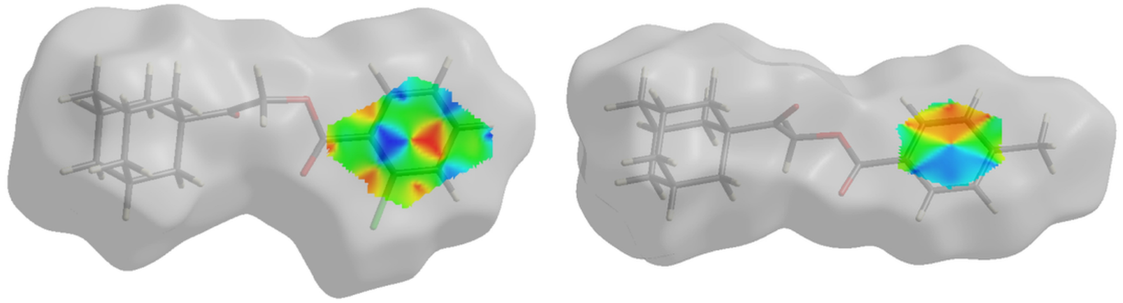

Figure S13. Hirshfeld surfaces of BUVCOM (left) and BUVDUT (right) mapped over shape-index showing the 'bow-tie' patterns of touching red and blue triangles depicting $\pi\cdots\pi$ interaction.

Tables

Table S1. Total electrostatic energies $E_{\text{tot}}^{\text{elec}}$ (kJ/mol)[a] for dimers involving different types of contacts.

Compound	Dimer	Interactions	E_{tot}	% [b]
BUVCIG	1	—	37.25	—
		H12D···O2A	3.70	9.93
		H4BA···Cg5	8.22	22.07
	2	—	31.07	—
		H6YA···Cg13	4.74	15.25
		H12B···O2B	6.29	20.24
BUVCOM	1	—	34.35	—
		H12C···O2A	4.67	13.60
		H4BA···Cg5	4.39	12.78
	2	—	26.17	—
		H18B···O3A	2.63	10.05
		H17B···Cl1A	5.16	19.72
		H9XA···Cl1B	3.93	15.02
BUVDUT	1	—	24.46	—
		H20C···O2	4.29	17.54
		Cg5···Cg5	5.45	22.28

[a] Calculated by Charger tool of MoProViewer.

[b] Percentage of the total electrostatic energy of the dimer.

Table S2. Enrichment ratios E_{XY} (reciprocal contacts merged) of intermolecular interactions.[a]

	**BUVCIG–Mol A**					**BUVCIG–Mol B**				
	H	C	O^a	O^b	O^c	H	C	O^a	O^b	O^c
H	1.01	—	—	—	—	0.89	—	—	—	—
C	0.91	1.49	—	—	—	1.24	0.52	—	—	—
O^a	1.06	0.85	0.00	—	—	1.34	0.29	0.00	—	—
O^b	1.32	0.33	0.00	0.00	—	1.37	0.15	0.00	0.00	—
O^c	1.13	0.90	0.00	0.00	0.00	1.32	0.36	0.00	0.00	0.00
	**BUVCOM–Mol A**					**BUVCOM–Mol B**				
	H	C	Cl	O^a	O^b	H	C	Cl	O^a	O^b
H	0.87	—	—	—	—	0.90	—	—	—	—
C	1.18	0.81	—	—	—	0.92	1.73	—	—	—
Cl	1.31	0.60	0.00	—	—	1.35	0.48	0.78	—	—
O^a	1.33	0.58	0.00	0.00	—	1.41	0.68	0.00	0.00	—
O^b	1.42	0.25	0.14	0.00	0.00	1.60	0.18	0.00	0.00	0.00
	**BUVCUS**					**BUVDAZ**				
	H	C	Cl	O^a	O^b	H	C	Cl	O^a	O^b
H	0.85	—	—	—	—	0.89	—	—	—	—
C	1.24	0.60	—	—	—	1.18	0.73	—	—	—
Cl	1.45	0.43	0.00	—	—	1.11	0.49	/	—	—
O^a	1.22	0.78	0.00	0.00	—	1.33	0.64	0.00	0.00	—
O^b	1.22	0.87	0.00	0.00	/	1.29	0.74	0.00	0.00	/
	**BUVDIH–Mol A**					**BUVDIH–Mol B**				
	H	C	O^a	O^b	O^c	H	C	O^a	O^b	O^c
H	1.00	—	—	—	—	0.92	—	—	—	—
C	0.88	1.71	—	—	—	1.18	0.59	—	—	—
O^a	1.18	0.75	0.00	—	—	1.20	1.20	1.20	1.20	1.20
O^b	1.33	0.14	0.00	0.00	—	1.28	0.36	0.00	0.00	—
O^c	1.11	1.03	0.00	0.00	0.00	1.37	0.00	0.00	0.00	0.00
	**BUVDON**					**BUVDUT**				
	H	C	O^a	O^b	O^c	H	C	O^a	O^b	O^c
H	0.98	—	—	—	—	0.99	—	—	—	—
C	1.09	0.66	—	—	—	0.91	1.61	—	—	—
O^a	1.10	0.75	0.00	—	—	1.10	0.93	/	—	—
O^b	1.15	0.72	0.00	/	—	1.38	0.03	0.00	0.00	—
O^c	0.94	0.54	/	0.00	0.00	1.19	0.80	0.00	0.00	0.00
	**BUTPUD**					**BUTQEO**				
	H	C	O^a	O^b	O^c	H	C	N	O^a	O^b
H	0.99	—	—	—	—	0.87	—	—	—	—
C	0.90	1.63	—	—	—	1.17	0.87	—	—	—
O^a	1.27	0.62	0.00	—	—	0.82	0.10	/	—	—
O^b	1.20	0.56	0.00	/	—	1.31	0.86	0.00	0.00	—
O^c	1.35	0.31	/	0.00	0.00	1.30	0.88	0.00	0.00	/

[a] E_{XY} values lower than 1.0% were not reported (/), as they are not meaningful. O^a, O^b, O^c, O^d, and O^e are oxygen ester O=C, oxygen ketone O=C, oxygen ester O–C, oxygen ether O–C, and oxygen O=N, respectively.

Table S3. Calculated electrostatic potential (V_s) (in kJ/mol) at the surface (isosurface 0.001 a.u) of *para*-substituted AOEB derivatives.

Compound	R	V^a	V^b	V^c	V^d	V^e	V_R^f
BUVCIG	H	-149.8	-159.0	-129.0	+90.5	-61.5	—
BUTQEO	NO_2	-107.4	-133.0	-104.7	+106.3	-5.9	-132.1
	—	—	—	—	—	—	-132.9
BUTPUD	OCH_3	-152.9	-163.6	-140.2	+84.5	-89.1	-105.4
BUVDUT	CH_3	-149.5	-160.9	-139.8	+87.9	-67.6	-90.3
BUVDAZ	Cl	-148.5	-129.5	-49.6	+101.5	-35.8	-71.8

[a]benzoate carbonyl oxygen; [b]carbonyl oxygen adjacent to adamantane; [c]ester ether oxygen; [d]methylene bridge; [e]phenyl ring face; [f]substituent group *R*.

Table S4. Topological properties (in a.u.) at the Bond Critical Point CP (3,−1) of selected intermolecular H···C interactions[a]

#CP	Interaction	$\rho \times 10^3$	$\nabla^2\rho \times 10^3$	$G(r) \times 10^3$	$V(r) \times 10^3$	$G(r)/\lvert V(r)\rvert$
**BUVCIG**						
58	H7YA···C19B	5.08	13.74	2.89	-2.35	1.23
82	H6YA···C16B	4.47	12.05	2.61	-2.20	1.18
66	H9BB···C19A	6.37	17.95	3.77	-3.06	1.23
85	H4BA···C16A	5.22	14.79	3.21	-2.72	1.18
**BUVCOM**						
60	H7B···C14	6.01	17.72	3.80	-3.17	1.20
**BUVCUS**						
67	H7YA···C19B	5.90	15.61	3.36	-2.81	1.20
52	H6YA···C16B	4.90	12.60	2.69	-2.22	1.21
**BUVDAZ**						
79	H7B···C17	4.98	13.37	2.88	-2.41	1.19
**BUVDIH**						
70	H7B···C14	5.53	15.02	3.22	-2.68	1.20
**BUVDON**						
76	H7YA···C19B	6.02	15.59	3.37	-2.83	1.19
65	H6YA···C16B	4.72	12.09	2.59	-2.15	1.20
**BUVDUT**						
83	H7B···C14	5.34	14.12	3.01	-2.49	1.21
**BUTPUD**						
87	H5A···C18	5.51	13.67	2.96	-2.50	1.18
79	H7A···C15	5.36	14.78	3.16	-2.63	1.20
**BUTQEO**						
78	H5A···C18	5.46	14.12	3.00	-2.46	1.22
77	H7A···C15	4.28	11.26	2.39	-1.97	1.22

[a] All values are in atomic units (a.u.). Factors of 10^3 are applied to the raw data for clarity. $G(r)$ is the kinetic energy density and $V(r)$ is the potential energy density.

Table S5. QTAIM dimerization energies (ΔE), H$\cdots$C distances, and H-bond binding energy (BE) involving methylene and methine H-atoms for AOEB with C–H$\cdots\pi$ interactions (kJ/mol, Å).

**Compound**	**Symmetry**	**Interactions**	ΔE	**H$\cdots$C**[a]	**BE**[b]	**H$\cdots$C**[c]	**BE**[d]
BUVCIG	$-1+x, 1+y, z$	H7YA$\cdots$C19B	−15.56	2.953	−1.40	—	—
		H6YA$\cdots$C17B		—	—	3.063	−1.03
	$1+x, y, z$	H9BB$\cdots$C19A	−17.03	2.850	−2.46	—	—
		H4BA$\cdots$C16A		—	—	2.973	−1.68
		H3BB$\cdots$C18A		3.073	−0.68	—	—
BUVCOM	x, y, z	H4BA$\cdots$C19B	−20.00	2.954	−1.67	—	—
		H5BB$\cdots$C18A		3.082	−0.51	—	—
		H4BA$\cdots$C19B		—	—	3.103	−0.78
BUVDIH	x, y, z	H9AA$\cdots$C14B	−18.79	2.996	−1.60	—	—
		H4AA$\cdots$C16B		—	—	3.102	−0.82
BUVDUT	$1-x, 1-y, -z$	H5B$\cdots$C18	−17.78	2.897	−1.75	—	—
		H7B$\cdots$C19		3.017	−1.05	—	—
BUTPUD	$-x, 2-y, -z$	H5A$\cdots$C18	−18.49	2.905	−2.03	—	—
		H7B$\cdots$C19		2.965	−1.90	—	—
Model-methylene	Vacuum	—	−12.1	—	—	—	—
Model-methine	Vacuum	—	−10.8	—	—	—	—

[a] Methylene H-atom. [b] H-bond binding energy for methylene H-atom. [c] Methine H-atom. [d] H-bond binding energy for methine H-atom.

Table S6. R factors and GoF values for five AOEB crystal structures

Compound	$R_{all}^{[a]}$	$R_{gt}^{[b]}$	$wR_{ref}^{[c]}$	$wR_{gt}^{[d]}$	GoF[e]
BUVCIG	0.0845	0.0650	0.2415	0.2159	1.031
BUVCOM	0.1599	0.0633	0.2155	0.1645	1.010
BUVDIH	0.0839	0.0652	0.2181	0.1943	1.027
BUVDUT	0.0639	0.0439	0.1311	0.1165	1.073
BUTPUD	0.0698	0.0538	0.1772	0.1576	1.041

[a] R_{all}: R-factor based on all reflections; [b] R_{gt}: R-factor for reflections with $I > 2\sigma(I)$; [c] wR_{ref}: weighted R-factor based on all reflections used in refinement; [d] wR_{gt}: weighted R-factor for reflections with $I > 2\sigma(I)$; [e] GoF: Goodness-of-Fit on F^2.

Table S7. Calculated energies and BSSE correction for five AOEB crystal structures

Compound	$E^{[a]}_{RB3LYP}$	$E^{[b]}_{CP}$	$E^{[c]}_{BSSE}$	$\sum E^{[d]}_{frag}$	$\Delta E^{[e]}_{raw}$	$\Delta E^{[f]}_{corr}$
BUVCIG	-264,091.95	-509,321.32	1.64	-509,317.25	-5.70	-4.07
BUVCOM	-552,488.08	-797,718.28	1.42	-797,713.51	-6.19	-4.78
BUVDIH	-288,762.99	-533,992.88	1.37	-533,988.40	-5.86	-4.49
BUVDUT	-288,769.49	-534,000.77	1.24	-533,996.52	-5.49	-4.25
BUTPUD	-335,953.60	-581,185.00	1.50	-581,180.57	-5.93	-4.42
Model-methylene	-264,094.37	-509,327.46	0.82	-509,324.85	-3.43	-2.61
Model-methine	-264,094.37	-509,327.36	0.64	-509,324.77	-3.23	-2.59

All energy values are reported in kcal/mol.
[a] E_{RB3LYP}: Total energy (RB3LYP level); [b] E_{CP}: Counterpoise corrected total energy; [c] E_{BSSE}: Basis Set Superposition Error energy; [d] $\sum E_{frag}$: Sum of individual fragment energies; [e] ΔE_{raw}: Complexation energy (raw); [f] ΔE_{corr}: Complexation energy (BSSE corrected).

Table S8. UFF optimized cartesian coordinates (Å) for Adamantane and Benzoic acid fragments

**Adamantane Fragment**				**Benzoic acid Fragment**			
Atom	X	Y	Z	Atom	X	Y	Z
C	-0.25501	-0.02097	1.04239	C	3.21010	0.54939	-0.03397
C	0.08724	-1.22430	0.14632	C	2.33534	-0.53244	0.06998
C	-1.02470	-1.44080	-0.89521	C	0.95573	-0.31713	0.08846
C	-2.35526	-1.70051	-0.16703	C	0.44963	0.98564	0.00262
C	-2.71143	-0.49987	0.72721	C	-1.00524	1.26536	0.01819
C	-2.83778	0.76071	-0.14633	C	1.33225	2.06835	-0.10173
C	-1.50995	1.03443	-0.87436	C	2.71063	1.84895	-0.11984
C	-0.39527	1.23689	0.16708	O	-1.52153	2.36545	-0.05234
C	-1.16062	-0.17490	-1.75956	O	-1.75192	0.15165	0.12171
C	-1.58995	-0.28871	1.75957	H	4.28389	0.37889	-0.04807
H	0.53958	0.12858	1.78175	H	2.72679	-1.54445	0.13681
H	1.04572	-1.05398	-0.35924	H	0.28517	-1.16896	0.16990
H	0.20640	-2.12590	0.75966	H	-2.67270	0.48770	0.11950
H	-0.77604	-2.29833	-1.53020	H	0.94836	3.08429	-0.16905
H	-3.15467	-1.87299	-0.89810	H	3.39343	2.69124	-0.20081
H	-2.27791	-2.61024	0.44099				
H	-3.65917	-0.69000	1.24301				
H	-3.64543	0.63039	-0.87707				
H	-3.10773	1.62233	0.47657				
H	-1.60546	1.93257	-1.49454				
H	-0.62342	2.10665	0.79537				
H	0.55496	1.44931	-0.33810				
H	-0.22350	0.01336	-2.29774				
H	-1.93960	-0.32122	-2.51787				
H	-1.49955	-1.17430	2.40053				
H	-1.83854	0.55495	2.41509				

Table S9. Selected NBO parameters for the C–H$\cdots\pi$ interactions in the experimental (BUVCIG) and idealized (Methylenic and Methinic) models.

**Model**	**Donor**	**Acceptor**	**Occup.**^{a}	$E^{(2)\ b}$
**BUVCIG**	$\pi(\mathrm{C{=}C})_{\mathrm{ring}}$	$\sigma^*(\mathrm{C–H})_{\mathrm{methy}}$	0.017	7.70
	$\pi(\mathrm{C{=}C})_{\mathrm{ring}}$	$\sigma^*(\mathrm{C–H})_{\mathrm{methi}}$	0.014	5.06
**Methylenic**	$\pi(\mathrm{C{=}C})_{\mathrm{ring}}$	$\sigma^*(\mathrm{C–H})_{\mathrm{methy}}$	0.015	6.53
**Methinic**	$\pi(\mathrm{C{=}C})_{\mathrm{ring}}$	$\sigma^*(\mathrm{C–H})_{\mathrm{methi}}$	0.012	4.10

^{a} Occupancy of the antibonding orbital σ^* (units in e^-).

^{b} Second-order perturbation stabilization energy in kJ/mol (1 kcal/mol = 4.184 kJ/mol).

Table S10. BOILED-Egg plot and SwissADME properties for BUVCIG

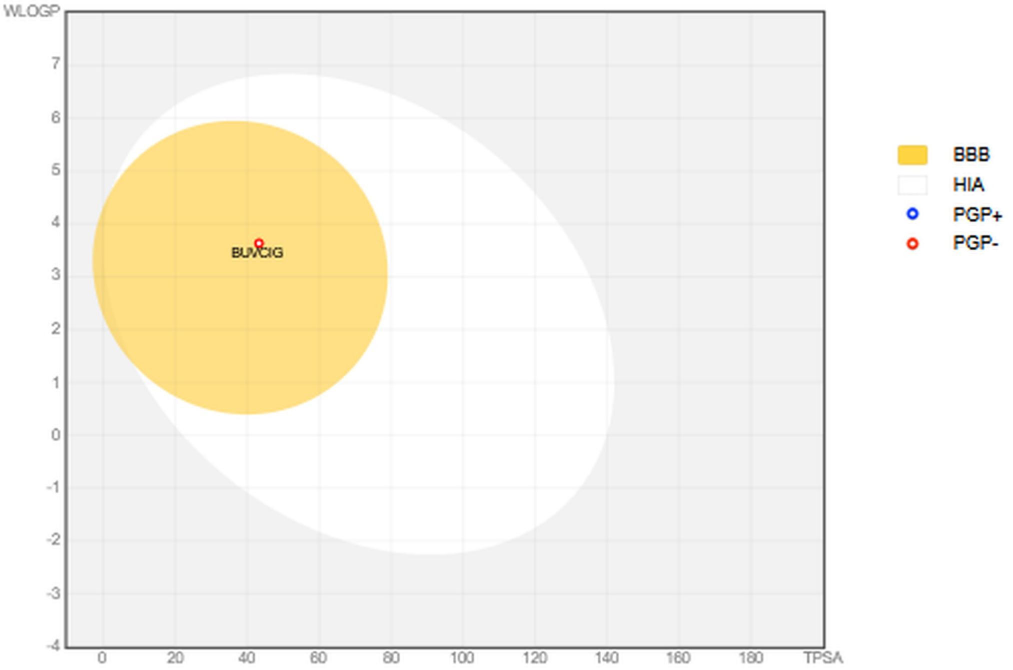

BUVCIG

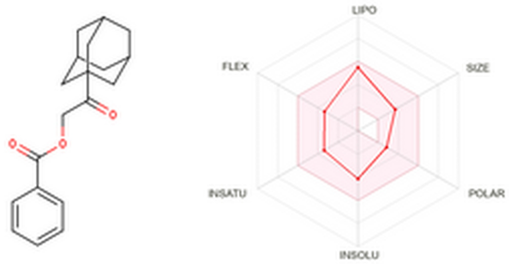

SMILES O=C(c1ccccc1)OCC(=O)C12CC3CC(C2)CC(C1)C3

Physicochemical Properties	
Formula	C19H22O3
Molecular weight	298.38 g/mol
Num. heavy atoms	22
Num. arom. heavy atoms	6
Fraction Csp3	0.58
Num. rotatable bonds	5
Num. H-bond acceptors	3
Num. H-bond donors	0
Molar Refractivity	84.20
TPSA	43.37 Å^2

Lipophilicity	
Log $P_{o/w}$ (iLOGP)	3.03
Log $P_{o/w}$ (XLOGP3)	4.11
Log $P_{o/w}$ (WLOGP)	3.63
Log $P_{o/w}$ (MLOGP)	3.52
Log $P_{o/w}$ (SILICOS-IT)	3.86
Consensus Log $P_{o/w}$	3.63

Water Solubility	
Log *S* (ESOL)	-4.15
Solubility	2.11e-02 mg/ml ; 7.06e-05 mol/l
Class	Moderately soluble
Log *S* (Ali)	-4.73
Solubility	5.60e-03 mg/ml ; 1.88e-05 mol/l
Class	Moderately soluble
Log *S* (SILICOS-IT)	-4.50
Solubility	9.52e-03 mg/ml ; 3.19e-05 mol/l
Class	Moderately soluble

Pharmacokinetics	
GI absorption	High
BBB permeant	Yes
P-gp substrate	No
CYP1A2 inhibitor	Yes
CYP2C19 inhibitor	Yes
CYP2C9 inhibitor	Yes
CYP2D6 inhibitor	Yes
CYP3A4 inhibitor	Yes
Log K_p (skin permeation)	-5.20 cm/s

Druglikeness	
Lipinski	Yes; 0 violation
Ghose	Yes
Veber	Yes
Egan	Yes
Muegge	Yes
Bioavailability Score	0.55

Medicinal Chemistry	
PAINS	0 alert
Brenk	0 alert
Leadlikeness	No; 1 violation: XLOGP3>3.5
Synthetic accessibility	4.45

Table S11. BOILED-Egg plot and SwissADME properties for BUVCOM

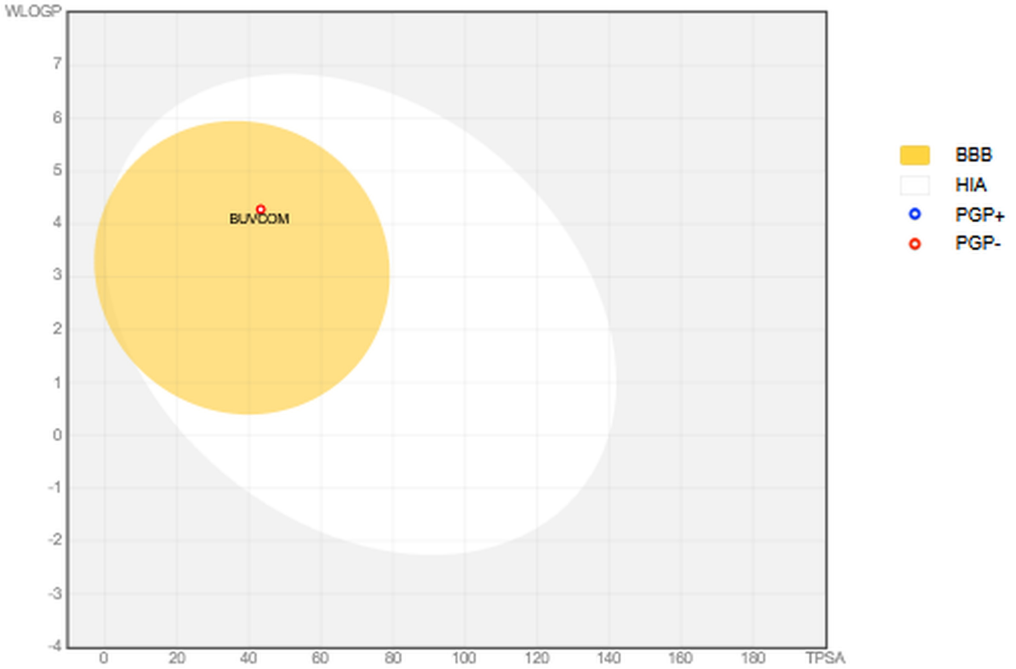

BUVCOM

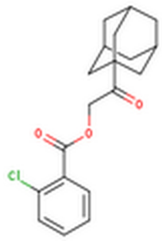

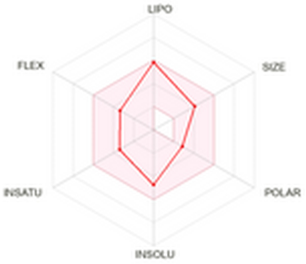

SMILES O=C(c1ccccc1Cl)OCC(=O)C12CC3CC(C2)CC(C1)C3

Physicochemical Properties	
Formula	C19H21ClO3
Molecular weight	332.82 g/mol
Num. heavy atoms	23
Num. arom. heavy atoms	6
Fraction Csp3	0.58
Num. rotatable bonds	5
Num. H-bond acceptors	3
Num. H-bond donors	0
Molar Refractivity	89.21
TPSA	43.37 Å²

Lipophilicity	
Log $P_{o/w}$ (iLOGP)	3.12
Log $P_{o/w}$ (XLOGP3)	4.73
Log $P_{o/w}$ (WLOGP)	4.28
Log $P_{o/w}$ (MLOGP)	4.02
Log $P_{o/w}$ (SILICOS-IT)	4.49
Consensus Log $P_{o/w}$	4.13

Water Solubility	
Log *S* (ESOL)	-4.75
Solubility	5.97e-03 mg/ml ; 1.79e-05 mol/l
Class	Moderately soluble
Log *S* (Ali)	-5.37
Solubility	1.42e-03 mg/ml ; 4.26e-06 mol/l
Class	Moderately soluble
Log *S* (SILICOS-IT)	-5.09
Solubility	2.69e-03 mg/ml ; 8.07e-06 mol/l
Class	Moderately soluble

Pharmacokinetics	
GI absorption	High
BBB permeant	Yes
P-gp substrate	No
CYP1A2 inhibitor	Yes
CYP2C19 inhibitor	Yes
CYP2C9 inhibitor	Yes
CYP2D6 inhibitor	Yes
CYP3A4 inhibitor	Yes
Log K_p (skin permeation)	-4.97 cm/s

Druglikeness	
Lipinski	Yes; 0 violation
Ghose	Yes
Veber	Yes
Egan	Yes
Muegge	Yes
Bioavailability Score	0.55

Medicinal Chemistry	
PAINS	0 alert
Brenk	0 alert
Leadlikeness	No; 1 violation: XLOGP3>3.5
Synthetic accessibility	4.65

Table S12. BOILED-Egg plot and SwissADME properties for BUVCUS

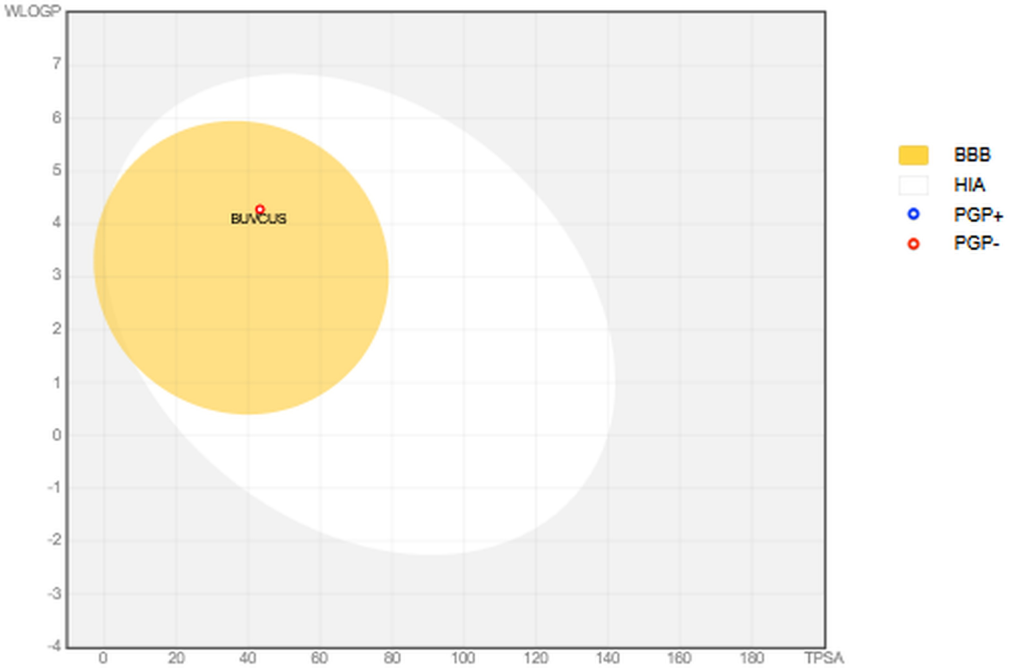

BUVCUS

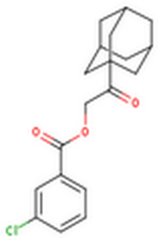

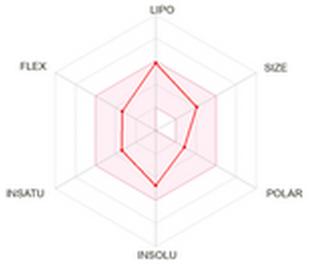

SMILES Clc1cccc(c1)C(=O)OCC(=O)C12CC3CC(C2)CC(C1)C3

Physicochemical Properties	
Formula	C19H21ClO3
Molecular weight	332.82 g/mol
Num. heavy atoms	23
Num. arom. heavy atoms	6
Fraction Csp3	0.58
Num. rotatable bonds	5
Num. H-bond acceptors	3
Num. H-bond donors	0
Molar Refractivity	89.21
TPSA	43.37 Å²

Lipophilicity	
Log $P_{o/w}$ (iLOGP)	3.34
Log $P_{o/w}$ (XLOGP3)	4.73
Log $P_{o/w}$ (WLOGP)	4.28
Log $P_{o/w}$ (MLOGP)	4.02
Log $P_{o/w}$ (SILICOS-IT)	4.49
Consensus Log $P_{o/w}$	4.17

Water Solubility	
Log *S* (ESOL)	-4.75
Solubility	5.97e-03 mg/ml ; 1.79e-05 mol/l
Class	Moderately soluble
Log *S* (Ali)	-5.37
Solubility	1.42e-03 mg/ml ; 4.26e-06 mol/l
Class	Moderately soluble
Log *S* (SILICOS-IT)	-5.09
Solubility	2.69e-03 mg/ml ; 8.07e-06 mol/l
Class	Moderately soluble

Pharmacokinetics	
GI absorption	High
BBB permeant	Yes
P-gp substrate	No
CYP1A2 inhibitor	Yes
CYP2C19 inhibitor	Yes
CYP2C9 inhibitor	Yes
CYP2D6 inhibitor	Yes
CYP3A4 inhibitor	Yes
Log K_p (skin permeation)	-4.97 cm/s

Druglikeness	
Lipinski	Yes; 0 violation
Ghose	Yes
Veber	Yes
Egan	Yes
Muegge	Yes
Bioavailability Score	0.55

Medicinal Chemistry	
PAINS	0 alert
Brenk	0 alert
Leadlikeness	No; 1 violation: XLOGP3>3.5
Synthetic accessibility	4.65

Table S13. BOILED-Egg plot and SwissADME properties for BUVDAZ

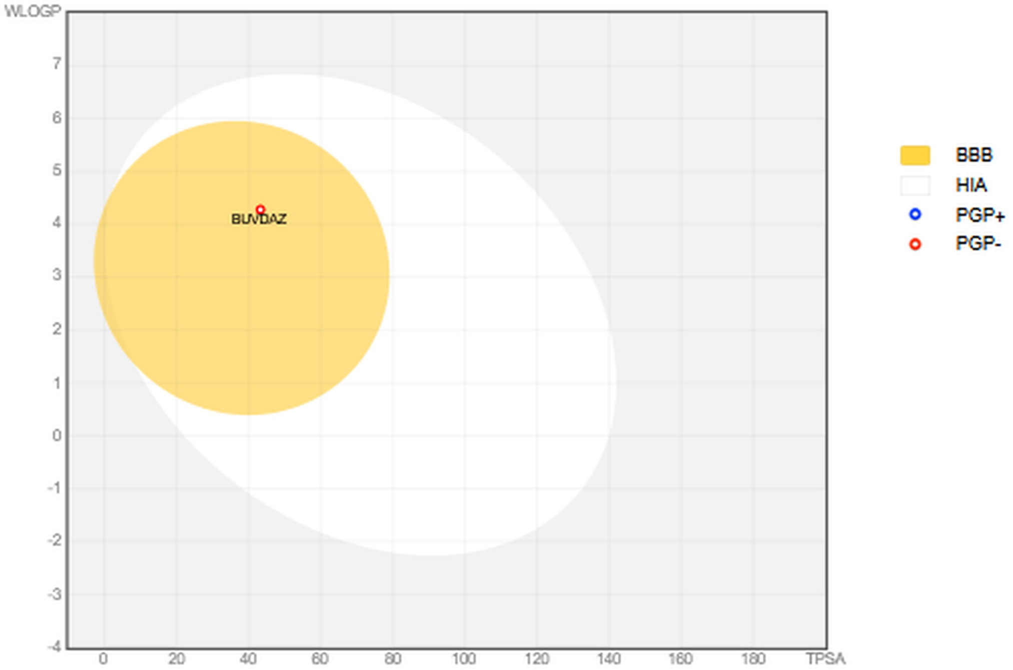

BUVDAZ

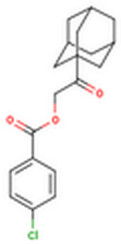

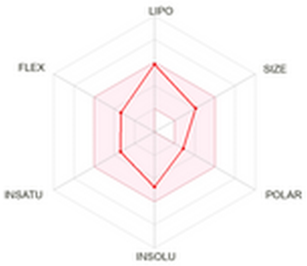

SMILES O=C(c1ccc(cc1)Cl)OCC(=O)C12CC3CC(C2)CC(C1)C3

Physicochemical Properties	
Formula	C19H21ClO3
Molecular weight	332.82 g/mol
Num. heavy atoms	23
Num. arom. heavy atoms	6
Fraction Csp3	0.58
Num. rotatable bonds	5
Num. H-bond acceptors	3
Num. H-bond donors	0
Molar Refractivity	89.21
TPSA	43.37 Å²

Lipophilicity	
Log $P_{o/w}$ (iLOGP)	3.38
Log $P_{o/w}$ (XLOGP3)	4.73
Log $P_{o/w}$ (WLOGP)	4.28
Log $P_{o/w}$ (MLOGP)	4.02
Log $P_{o/w}$ (SILICOS-IT)	4.49
Consensus Log $P_{o/w}$	4.18

Water Solubility	
Log *S* (ESOL)	-4.75
Solubility	5.97e-03 mg/ml ; 1.79e-05 mol/l
Class	Moderately soluble
Log *S* (Ali)	-5.37
Solubility	1.42e-03 mg/ml ; 4.26e-06 mol/l
Class	Moderately soluble
Log *S* (SILICOS-IT)	-5.09
Solubility	2.69e-03 mg/ml ; 8.07e-06 mol/l
Class	Moderately soluble

Pharmacokinetics	
GI absorption	High
BBB permeant	Yes
P-gp substrate	No
CYP1A2 inhibitor	Yes
CYP2C19 inhibitor	Yes
CYP2C9 inhibitor	Yes
CYP2D6 inhibitor	Yes
CYP3A4 inhibitor	Yes
Log K_p (skin permeation)	-4.97 cm/s

Druglikeness	
Lipinski	Yes; 0 violation
Ghose	Yes
Veber	Yes
Egan	Yes
Muegge	Yes
Bioavailability Score	0.55

Medicinal Chemistry	
PAINS	0 alert
Brenk	0 alert
Leadlikeness	No; 1 violation: XLOGP3>3.5
Synthetic accessibility	4.54

Table S14. BOILED-Egg plot and SwissADME properties for BUVDIH

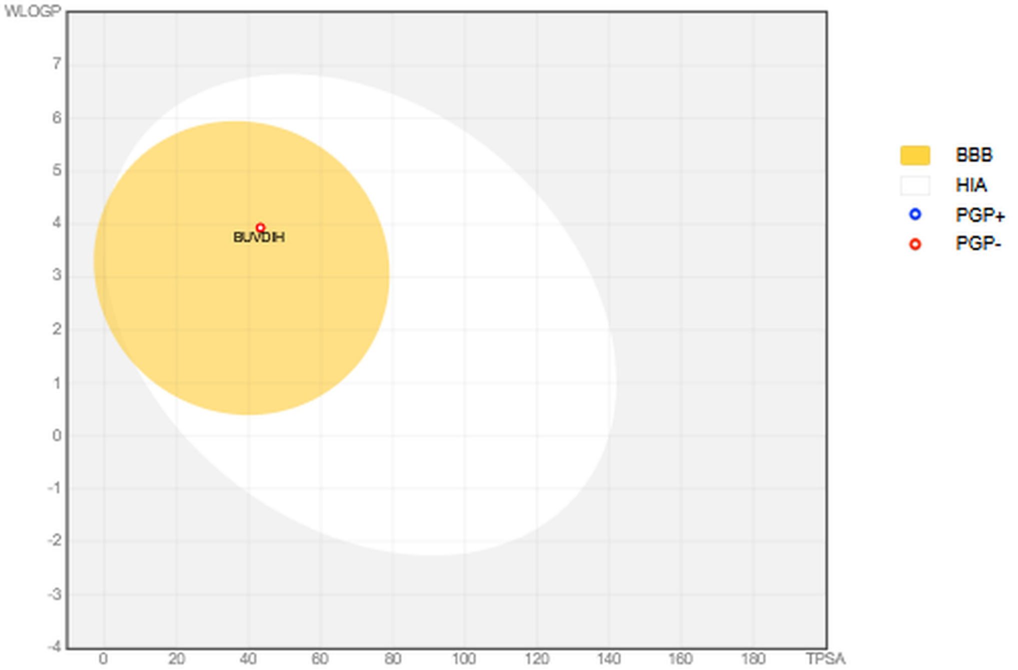

BUVDIH

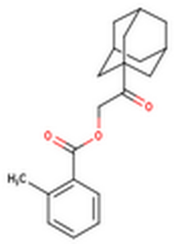

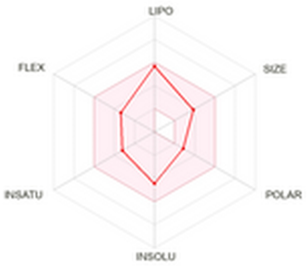

SMILES O=C(c1ccccc1C)OCC(=O)C12CC3CC(C2)CC(C1)C3

Physicochemical Properties	
Formula	C20H24O3
Molecular weight	312.40 g/mol
Num. heavy atoms	23
Num. arom. heavy atoms	6
Fraction Csp3	0.60
Num. rotatable bonds	5
Num. H-bond acceptors	3
Num. H-bond donors	0
Molar Refractivity	89.16
TPSA	43.37 Å²

Lipophilicity	
Log $P_{o/w}$ (iLOGP)	3.37
Log $P_{o/w}$ (XLOGP3)	4.47
Log $P_{o/w}$ (WLOGP)	3.94
Log $P_{o/w}$ (MLOGP)	3.75
Log $P_{o/w}$ (SILICOS-IT)	4.37
Consensus Log $P_{o/w}$	3.98

Water Solubility	
Log *S* (ESOL)	-4.46
Solubility	1.09e-02 mg/ml ; 3.50e-05 mol/l
Class	Moderately soluble
Log *S* (Ali)	-5.10
Solubility	2.48e-03 mg/ml ; 7.93e-06 mol/l
Class	Moderately soluble
Log *S* (SILICOS-IT)	-4.88
Solubility	4.16e-03 mg/ml ; 1.33e-05 mol/l
Class	Moderately soluble

Pharmacokinetics	
GI absorption	High
BBB permeant	Yes
P-gp substrate	No
CYP1A2 inhibitor	No
CYP2C19 inhibitor	Yes
CYP2C9 inhibitor	Yes
CYP2D6 inhibitor	Yes
CYP3A4 inhibitor	Yes
Log K_p (skin permeation)	-5.03 cm/s

Druglikeness	
Lipinski	Yes; 0 violation
Ghose	Yes
Veber	Yes
Egan	Yes
Muegge	Yes
Bioavailability Score	0.55

Medicinal Chemistry	
PAINS	0 alert
Brenk	0 alert
Leadlikeness	No; 1 violation: XLOGP3>3.5
Synthetic accessibility	4.67

Table S15. BOILED-Egg plot and SwissADME properties for BUVDON

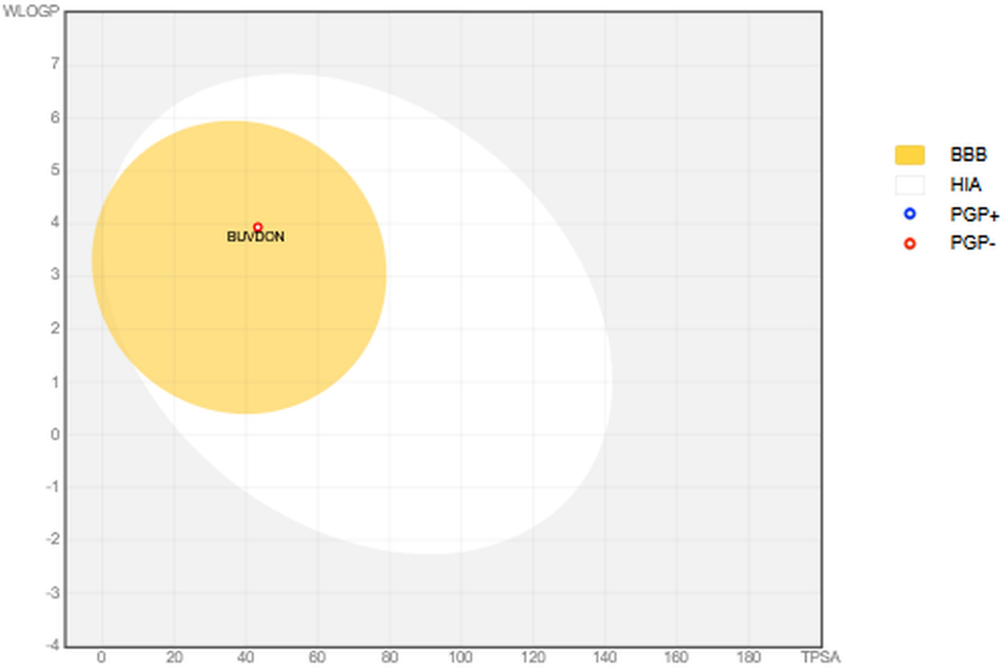

BUVDON

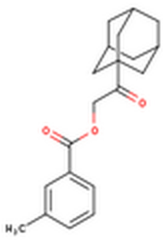

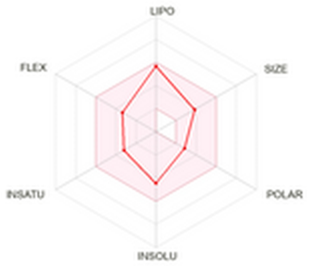

SMILES Cc1cccc(c1)C(=O)OCC(=O)C12CC3CC(C2)CC(C1)C3

Physicochemical Properties	
Formula	C20H24O3
Molecular weight	312.40 g/mol
Num. heavy atoms	23
Num. arom. heavy atoms	6
Fraction Csp3	0.60
Num. rotatable bonds	5
Num. H-bond acceptors	3
Num. H-bond donors	0
Molar Refractivity	89.16
TPSA	43.37 Å²

Lipophilicity	
Log $P_{o/w}$ (iLOGP)	3.19
Log $P_{o/w}$ (XLOGP3)	4.47
Log $P_{o/w}$ (WLOGP)	3.94
Log $P_{o/w}$ (MLOGP)	3.75
Log $P_{o/w}$ (SILICOS-IT)	4.37
Consensus Log $P_{o/w}$	3.94

Water Solubility	
Log *S* (ESOL)	-4.46
Solubility	1.09e-02 mg/ml ; 3.50e-05 mol/l
Class	Moderately soluble
Log *S* (Ali)	-5.10
Solubility	2.48e-03 mg/ml ; 7.93e-06 mol/l
Class	Moderately soluble
Log *S* (SILICOS-IT)	-4.88
Solubility	4.16e-03 mg/ml ; 1.33e-05 mol/l
Class	Moderately soluble

Pharmacokinetics	
GI absorption	High
BBB permeant	Yes
P-gp substrate	No
CYP1A2 inhibitor	No
CYP2C19 inhibitor	Yes
CYP2C9 inhibitor	Yes
CYP2D6 inhibitor	Yes
CYP3A4 inhibitor	Yes
Log K_p (skin permeation)	-5.03 cm/s

Druglikeness	
Lipinski	Yes; 0 violation
Ghose	Yes
Veber	Yes
Egan	Yes
Muegge	Yes
Bioavailability Score	0.55

Medicinal Chemistry	
PAINS	0 alert
Brenk	0 alert
Leadlikeness	No; 1 violation: XLOGP3>3.5
Synthetic accessibility	4.70

Table S16. BOILED-Egg plot and SwissADME properties for BUVDUT

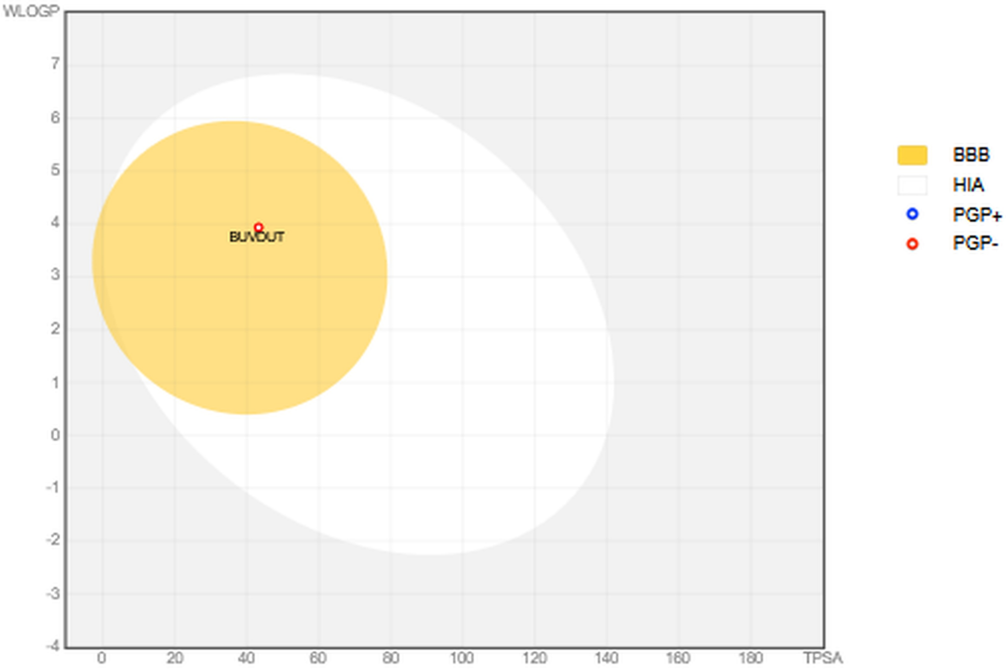

BUVDUT

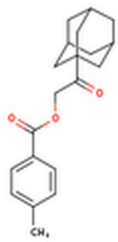

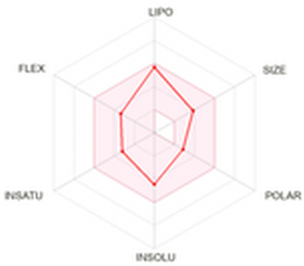

SMILES O=C(c1ccc(cc1)C)OCC(=O)C12CC3CC(C2)CC(C1)C3

Physicochemical Properties	
Formula	C20H24O3
Molecular weight	312.40 g/mol
Num. heavy atoms	23
Num. arom. heavy atoms	6
Fraction Csp3	0.60
Num. rotatable bonds	5
Num. H-bond acceptors	3
Num. H-bond donors	0
Molar Refractivity	89.16
TPSA	43.37 Å²

Lipophilicity	
Log $P_{o/w}$ (iLOGP)	3.30
Log $P_{o/w}$ (XLOGP3)	4.47
Log $P_{o/w}$ (WLOGP)	3.94
Log $P_{o/w}$ (MLOGP)	3.75
Log $P_{o/w}$ (SILICOS-IT)	4.37
Consensus Log $P_{o/w}$	3.97

Water Solubility	
Log *S* (ESOL)	-4.46
Solubility	1.09e-02 mg/ml ; 3.50e-05 mol/l
Class	Moderately soluble
Log *S* (Ali)	-5.10
Solubility	2.48e-03 mg/ml ; 7.93e-06 mol/l
Class	Moderately soluble
Log *S* (SILICOS-IT)	-4.88
Solubility	4.16e-03 mg/ml ; 1.33e-05 mol/l
Class	Moderately soluble
**Pharmacokinetics**	
GI absorption	High
BBB permeant	Yes
P-gp substrate	No
CYP1A2 inhibitor	No
CYP2C19 inhibitor	Yes
CYP2C9 inhibitor	Yes
CYP2D6 inhibitor	Yes
CYP3A4 inhibitor	Yes
Log K_p (skin permeation)	-5.03 cm/s
**Druglikeness**	
Lipinski	Yes; 0 violation
Ghose	Yes
Veber	Yes
Egan	Yes
Muegge	Yes
Bioavailability Score	0.55
**Medicinal Chemistry**	
PAINS	0 alert
Brenk	0 alert
Leadlikeness	No; 1 violation: XLOGP3>3.5
Synthetic accessibility	4.62

Table S17. BOILED-Egg plot and SwissADME properties for BUTPUD

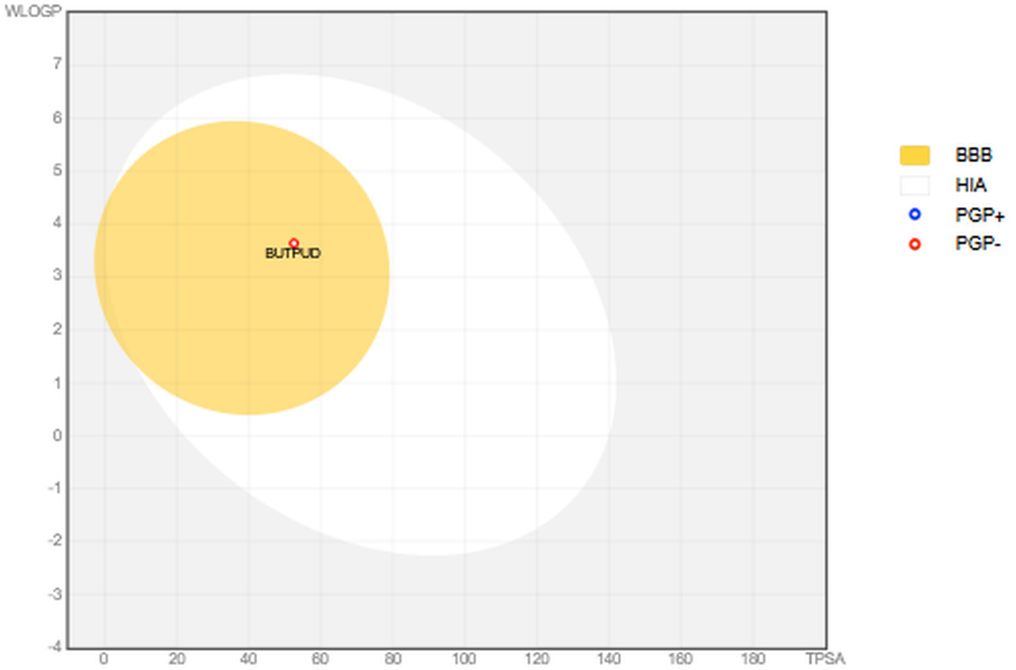

BUTPUD

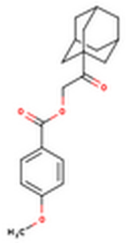

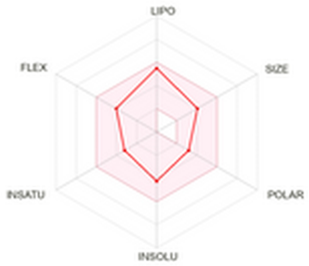

SMILES COc1ccc(cc1)C(=O)OCC(=O)C12CC3CC(C2)CC(C1)C3

Physicochemical Properties	
Formula	C20H24O4
Molecular weight	328.40 g/mol
Num. heavy atoms	24
Num. arom. heavy atoms	6
Fraction Csp3	0.60
Num. rotatable bonds	6
Num. H-bond acceptors	4
Num. H-bond donors	0
Molar Refractivity	90.69
TPSA	52.60 Å²

Lipophilicity	
Log $P_{o/w}$ (iLOGP)	3.47
Log $P_{o/w}$ (XLOGP3)	4.08
Log $P_{o/w}$ (WLOGP)	3.64
Log $P_{o/w}$ (MLOGP)	3.17
Log $P_{o/w}$ (SILICOS-IT)	3.90
Consensus Log $P_{o/w}$	3.65

Water Solubility	
Log *S* (ESOL)	-4.24
Solubility	1.91e-02 mg/ml ; 5.81e-05 mol/l
Class	Moderately soluble
Log *S* (Ali)	-4.89
Solubility	4.23e-03 mg/ml ; 1.29e-05 mol/l
Class	Moderately soluble
Log *S* (SILICOS-IT)	-4.61
Solubility	8.10e-03 mg/ml ; 2.47e-05 mol/l
Class	Moderately soluble

Pharmacokinetics	
GI absorption	High
BBB permeant	Yes
P-gp substrate	No
CYP1A2 inhibitor	No
CYP2C19 inhibitor	Yes
CYP2C9 inhibitor	Yes
CYP2D6 inhibitor	Yes
CYP3A4 inhibitor	Yes
Log K_p (skin permeation)	-5.41 cm/s

Druglikeness	
Lipinski	Yes; 0 violation
Ghose	Yes
Veber	Yes
Egan	Yes
Muegge	Yes
Bioavailability Score	0.55

Medicinal Chemistry	
PAINS	0 alert
Brenk	0 alert
Leadlikeness	No; 1 violation: XLOGP3>3.5
Synthetic accessibility	4.61

Table S18. BOILED-Egg plot and SwissADME properties for BUTQEO

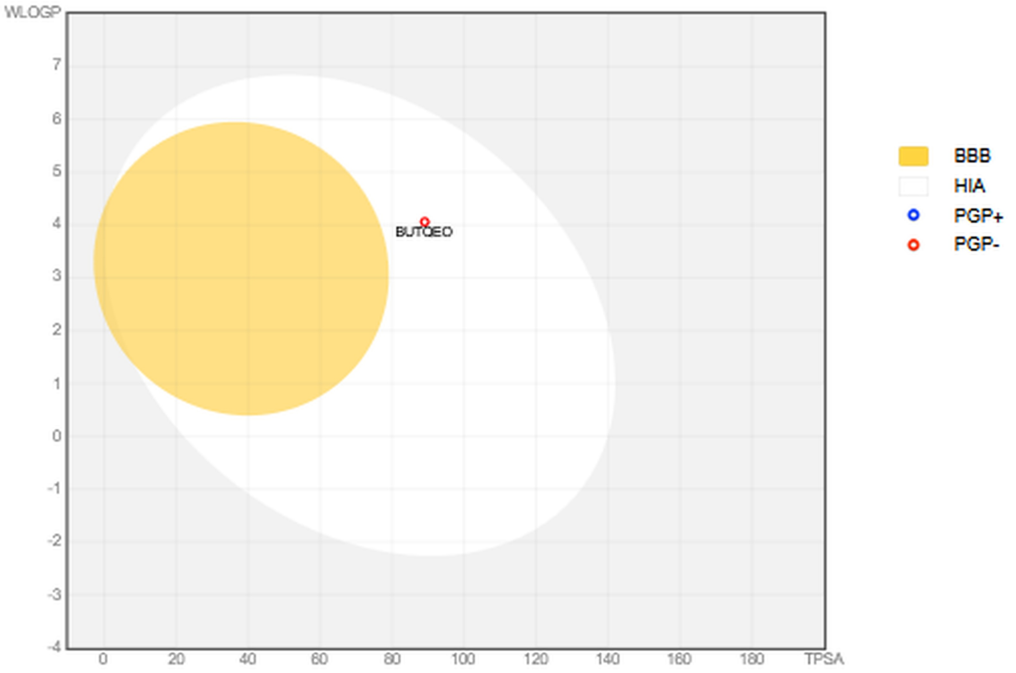

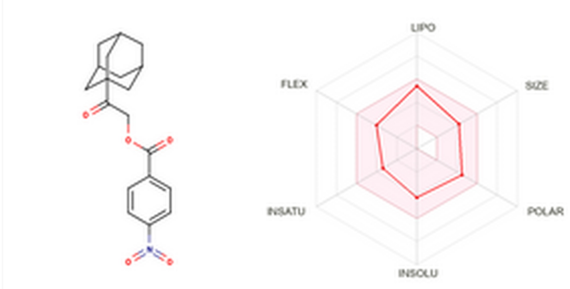

SMILES O=C(c1ccc(cc1)N(=O)=O)OCC(=O)C12CC3CC(C2)CC(C1)C3

Physicochemical Properties	
Formula	C19H21NO5
Molecular weight	343.37 g/mol
Num. heavy atoms	25
Num. arom. heavy atoms	6
Fraction Csp3	0.58
Num. rotatable bonds	6
Num. H-bond acceptors	5
Num. H-bond donors	0
Molar Refractivity	93.02
TPSA	89.19 Å²

Lipophilicity	
Log $P_{o/w}$ (iLOGP)	2.24
Log $P_{o/w}$ (XLOGP3)	3.94
Log $P_{o/w}$ (WLOGP)	4.06
Log $P_{o/w}$ (MLOGP)	2.42
Log $P_{o/w}$ (SILICOS-IT)	2.08
Consensus Log $P_{o/w}$	2.95

Water Solubility	
Log *S* (ESOL)	-4.23
Solubility	2.01e-02 mg/ml ; 5.85e-05 mol/l
Class	Moderately soluble
Log *S* (Ali)	-5.51
Solubility	1.05e-03 mg/ml ; 3.07e-06 mol/l
Class	Moderately soluble
Log *S* (SILICOS-IT)	-4.33
Solubility	1.61e-02 mg/ml ; 4.68e-05 mol/l
Class	Moderately soluble

Pharmacokinetics	
GI absorption	High
BBB permeant	No
P-gp substrate	No
CYP1A2 inhibitor	No
CYP2C19 inhibitor	Yes
CYP2C9 inhibitor	Yes
CYP2D6 inhibitor	No
CYP3A4 inhibitor	Yes
Log K_p (skin permeation)	-5.60 cm/s

Druglikeness	
Lipinski	Yes; 0 violation
Ghose	Yes
Veber	Yes
Egan	Yes
Muegge	Yes
Bioavailability Score	0.55

Medicinal Chemistry	
PAINS	0 alert
Brenk	1 alert: nitro_group
Leadlikeness	No; 1 violation: XLOGP3>3.5
Synthetic accessibility	4.64
